# Biochemical and Molecular Investigation of the Effect of Saponins and Terpenoids Derived from Leaves of *Ilex aquifolium* on Lipid Metabolism of Obese Zucker Rats

**DOI:** 10.3390/molecules27113376

**Published:** 2022-05-24

**Authors:** Natalia Pachura, Robert Kupczyński, Kamila Lewandowska, Maciej Włodarczyk, Marta Klemens, Piotr Kuropka, Renata Nowaczyk, Małgorzata Krzystek-Korpacka, Iwona Bednarz-Misa, Tomasz Sozański, Krystyna Pogoda-Sewerniak, Antoni Szumny

**Affiliations:** 1 Faculty of Biotechnology and Food Science, Wrocław University of Environmental and Life Sciences, Norwida 25, 50-375 Wroclaw, Poland; 112002@student.upwr.edu.pl (M.K.); antoni.szumny@upwr.edu.pl (A.S.); 2 Department of Environment, Animal Hygiene and Welfare, Wrocław University of Environmental and Life Sciences, Chełmońskiego 38C, 51-630 Wroclaw, Poland; 100364@student.upwr.edu.pl (K.L.); krystyna.pogoda-sewerniak@upwr.edu.pl (K.P.-S.); 3 Department of Pharmacognosy and Herbal Medicines, Faculty of Pharmacy, Wroclaw Medical University, Borowska 211a, 50-556 Wroclaw, Poland; maciej.wlodarczyk@umw.edu.pl; 4 Department of Animal Physiology and Biostructure, Wrocław University of Environmental and Life Sciences, Norwida 31, 50-375 Wroclaw, Poland; piotr.kuropka@upwr.edu.pl (P.K.); renata.nowaczyk@upwr.edu.pl (R.N.); 5 Department of Pharmacology, Wroclaw Medical University, ul. J. Mikulicza-Radeckiego 2, 50-345 Wrocław, Poland; malgorzata.krzystek-korpacka@umw.edu.pl (M.K.-K.); iwona.bednarz-misa@umw.edu.pl (I.B.-M.); tomasz.sozanski@umw.edu.pl (T.S.)

**Keywords:** terpenoids, saponins, Zucker rats, lipid metabolism

## Abstract

*Ilex paraguariensis*, the holly tree, is a plant with recognized biological properties, whose aqueous infusions are known as “Yerba mate”, that regulate lipid metabolism, reduce obesity, and improve brain stimulation. In the present study, the effect of standardized saponin and terpenoid fractions of a European taxon, *Ilex aquifolium*, on blood biochemical parameters in a rat model of metabolic disorder, (fa/fa) Zucker, are presented. The profiles of the volatile fractions of two species and six European varieties of *Ilex* were investigated. After selecting the best variety, the saponin and terpenoid fractions were isolated and standardized, and animals were fed 10 mg kg^−1^ b.w. for 8 weeks. A statistically significant decrease in liver adiposity was observed, confirmed by histology and quantitative identification (gas chromatography–mass spectrometry analyses of hepatic lipids. RT-qPCR analysis of gene expression in the aorta revealed that the administration of the terpenoid fraction downregulated *LOX-1*, suggesting a reduction in atherosclerotic stimuli. In addition, a statistically significant reduction (*p* < 0.05) in *PPARγ* for the saponin fraction was observed in the liver. The expression of the *ACAT-1* gene in the liver, responsible for the formation of cholesterol esters, increased significantly in the group receiving the terpenoid fraction compared to the control, which was also confirmed by the analysis of individual blood biochemical parameters. The opposite effect was observed for saponins. Taking the above into account, it is shown for the first time that *Ilex aquifolium* can be a source of compounds that positively influence lipid metabolism.

## 1. Introduction

Metabolic disorders such as hepatic steatosis represent a growing problem in modern populations. In addition to classical pharmacotherapy, so-called herbal medicines or dietary supplements that regulate the lipid metabolism of the organism may constitute an alternative. Currently, the most widely used herbal drugs are extracts containing flavonolignan silymarin from *Silybum marianum* and the derivative hydroxycinnamic acid cynarin of *Cynara scolymus*, which are used in the treatment of liver damage; they protect the liver from toxic compounds and improve its regeneration. These compounds also exert spasmolytic and cholagogic effects [1]. According to Speroni et al., artichoke leaf extract shows protective effects against oxidative stress in hepatocytes [2]. Lipid-regulating herbs also include those rich in saponin fractions, e.g., *Momordica charantia*, *Gymnema sylvestre*, *Gypsophila oldhamiana*, or *Achyranthes aspera*. Their mechanisms of action range from appetite regulation, and the inhibition of pancreatic lipase to effects on triglyceride absorption, retrosynthesis, and adipogenesis [3].

In recent years, interest in the traditional South American beverage of “Yerba mate”, the water macerate of *Ilex paraguariensis*, has grown tremendously, which has resulted in a significant increase in research and literature on its biological properties. The active components responsible for its biological activities are: polyphenols, saponins, triterpenoids, and finally caffeine. These have exhibited effects such as vasodilation and lipid lowering, anti-glycation, and anti-obesity effects [4]. In addition, *I. paraquariensis* has mutagenic or antimutagenic properties, depending on the model. It affects the stimulation of the nervous system and has diuretic properties. “Yerba mate” was also found to protect DNA from oxidation and low-density lipoprotein lipoperoxidation in vitro [5]. The methanolic extracts of the leaves present high antioxidant activity and phenolic compound content such as chlorogenic acid derivatives, flavonoids, and saponins [6]. In 2020, Zapata [7] published results (obtained on a Wistar model with a high-fat and high-sugar diet) indicating that only caffeine is responsible for suppressing lipid accumulation, proven by its modulation of gene expression. Caffeine-free, supercritical CO_2_ extracts of *I. paraguariensis* did not affect fatty acid synthase (Fasn), pyruvate kinase or the microsomal triglyceride transfer protein.

The European varieties of *Ilex aquifolium* and *Ilex meserveae* have not yet been as thoroughly characterized as the South American *I. paraguariensis* for their biochemicals and activities. Our previous experience indicates that, despite geographical separation, the chemical composition of the European and South American *Ilex* varieties is similar. The *I. aquifolium* and *I. meserveae* cultivars are, as with *I. paraguariensis*, rich in polyphenols, saponins, and triterpenoid fractions (with derivatives of ursolic acid, oleanolic acid, etc.). The main difference in chemotype is the complete absence of the xanthine fraction, i.e., caffeine or theobromine. In the context of the publication by Zapata et al. [7], where it was suggested that for the *paraguariensis*, the only active fraction (for regulation of lipid metabolism) is xanthines, it seems reasonable to test decaffeinated European varieties. Our preliminary study confirmed the renal protective effect for a high-cholesterol diet of aqueous extracts and saponin and terpenoid fractions of the *I. aquifolium* variety on a Wistar model [8]. Therefore, it seems reasonable to test whether decaffeinated *I. aquifolium* extracts, as well as saponin and terpenoid fractions, will have a protective effect on the metabolism of the Zucker knockout rat. The purpose of this study was to determine whether the fractions of *I. aquifolium* would alter the histopathological and biochemical picture of the Zucker rat, as well as to determine changes in the expression of selected genes responsible for normal body metabolism.

## 2. Results

### 2.1. Aroma Profile of Ilex

This is the first paper to present a comparison of the volatile compound profiles in Argentine *I. paraguariensis* and European *I. aquifolium* and *I. meserveae*. The expected results of the study of the volatile fractions of European holly species were the appearance of rich monoterpene and monoterpenoid profiles, which have not yet undergone oxidation reactions during heat treatment. The analysis of volatile compounds in different varieties of *Ilex* revealed a total of 81 compounds, of which the ten main ones are presented in Table 1 (in addition, Table S1 showing all compounds is available in the Supplementary Data ). The results obtained show a definite difference in the volatile compound profile between Argentine *I. paraguariensis* and European cultivars of *I. aquifolium* and *I. meserveae*. This difference was due to the fact that *I. paraguariensis* was subjected to a prior drying process that contributed to the formation of more oxidation intermediates, characterized by a lower boiling point. The profiles presented by the different groups can represent the characteristic distribution of the chemotype within a given cultivar and act as specific marker indicators to assist in the identification of individual holly species. A divergence was also observed among the profiles of European cultivars. In *I. paraguariensis*, the main volatile compounds were found to be 6-methyl-5-heptene-2-one (25.73 µg g^−1^), (*E*,*E*)-3,5-octadien-2-one (22.82 µg g^−1^), (*E*,*E*)-2,4-heptadienal (14.83 µg g^−1^), hexanal (13.80 µg g^−1^) and 2-methyl-2-pentenal (11.77 µg g^−1^). The two main compounds found in *I. paraguariensis* (6-methyl-5-heptene-2-one and (*E*,*E*)-3,5-octadien-2-one) were absent in the European varieties. In *I. aquifolium*, (*E*,*E*)-2,4-heptadienal was also absent. and p-cymene (58.21–82.62 µg g^−1^), α-phellandrene (3.68–5.35 µg g^−1^) and α-pinene (1.98–3.94 µg g^−1^) were the main compounds. In *I. meserveae*, p-cymene (20.20–32.75 µg g^−1^) was also among the main compounds, but the other compounds were (*E*)-2-hexenal (12.28–20.97 µg g^−1^) and 3-hexen-1-ol (6.34–11.85 µg g^−1^).

Regarding the profile of volatile compounds in *I. paraguariensis*, this has already been reported in several works. A study by Dallago et al. [9] compared the profile of volatile compounds contained in *I. paraguariensis* both before the drying process (fresh green leaves) and after the drying stage. The results presented differences in the number of Maillard reaction products formed and their percentage ratios. It was observed that the effect of a higher temperature and oxygen access favored the formation of more secondary products such as ketones, aldehydes, and carboxylic acids. Fresh leaf samples were found to be dominated by compounds from the alcohol group, while dry leaf samples were dominated by aldehydes and ketones. It was also found that long hydrocarbon chains are degraded first, followed by the oxidation of individual functional groups. One paper that shows values similar to those of our team in the profile of volatile compounds of *I. paraguariensis* is the publication by Araujo et al. [10]. They showed that the main aromatic compounds in mate are (*E*,*E*)-2,4-heptadienal (7.8%), (*E*,*Z*)-2,4-heptadienal (7.0%) and (*E*,*Z*)-3,5-octadien-2-one (5.2%). However, they obtained a much lower value for 6-methyl-5-hepten-2-one (1.4%), which in our study, proved to be the main component of the volatile profile. In a study by Marquez et al. [11], they observed that geranyl acetate (9.38 µg g^−1^), linalool (4.78 µg g^−1^) and isomers (*E*,*E*)- and (*E*,*Z*)-2,4-heptadienal (3.60 and 1.21 µg g^−1^) were the most abundant in Yerba mate leaves. Conversely, Martins et al. [12] conducted a study on the volatile compounds released in relation to mechanical damage and herbivore exposure. In the control group, which was *I. paraguariensis*, the main volatile compounds reported were decanal, nonanal, and limonene.

### 2.2. Terpenoid Profile

The analysis of terpenoids from two cultivars of European *Ilex*, i.e., *I. aquifolium* and *I. meserveae*, showed the presence of 12 compounds, of which the seven main ones are presented in Table 2 (in addition, Table S2 showing all compounds is available in the Supplementary Data). The study showed similarities in the terpenoid profiles of the European cultivars. The dominant compounds in both *I. aquifolium* and *I. meserveae* were ursolic acid (7.29–16.11 mg g^−1^), oleanolic acid (2.03–6.62 mg g^−1^), α-amyrin (0.36–3.22 mg g^−1^), lupeol (0.42–2.53 mg g^−1^) and uvaol (0.37–2.48 mg g^−1^). For comparison, the reference sample was *I. paraguariensis*, whose terpenoid profile was similar to those of the European varieties, with the difference being that a significantly lower content of ursolic acid (1.23 mg g^−1^) was observed in Yerba mate leaves.

Previous studies have also reported the presence of tritepenes in other *Ilex* cultivars. Lupeol, betulonic acid, uvaol, ursolic acid, and α-amyrin have been identified in the leaves of *I. cornuta* and *I. latifolia* [13]. Compounds from the terpenoid group were also documented in *I. centrochinensis* and *I. macropoda*. In the first species, the presence of lupeol and oleanolic acid was revealed, and in the second, betulin was isolated in addition to lupeol [14]. Plants with a high content of pentacyclic tritepenes are often used in phytotherapy due to their valuable medicinal properties. Additionally, they are widely distributed in the plant world and are the subject of phytochemical and pharmacological research. One of them is *Ficus carica*, a traditional plant in folk medicine used to combat pneumonia, diarrhea, inflammation, and indigestion. Ivanov et al. [15] began to identify the constituents of the nonpolar fraction using GC-MS, and the results of their study show that pentacyclic triterpenoids α-Amyrin, β-amyrin and lupeol are present in ficus leaves. Wolbiś et al. [16], in their study of the quats and leaves of *Prunus spinosa*, which is known as a tea substitute, proved that more triterpene acids are present, and these are mainly ursolic acid (9.5 mg g^−1^) and oleanolic acid (2.3 mg g^−1^). Conversely, Kowalski [17] obtained similar values for ursolic acid (14.98 mg g^−1^) and oleanolic acid (approximately 5 mg g^−1^) [17] in the leaves of *Silphium integrifolium*, whose infusion was used to treat urinary tract disorders. Cheun and Zhang [18], in *Prunella vulgaris* spikes, identified mainly betulinic acid, ursolic acid, and oleanolic acid, whose amounts were 4.7, 4.0 and 0.9 mg g^−1^, respectively. A terpenoid fraction was also isolated from the herb *Hieracium pilosella* [19], used, in the treatment of skin diseases, among others, due to its astringent, antiseptic, and anti-inflammatory activities. This fraction consists of β-amyrin, lupeol, and α-amyrin. In conclusion, terpenoids are a widely distributed group among medicinal plants that show promise for the development of new, multifunctional bioactive agents.

### 2.3. Saponin Profile

Following our previous UHPLC-MS/MS analyses, the saponin profiles of *I. aquifolium* and *I. paraguariensis* were compared under the conditions described above [8]. By comparing the saponin profiles of holly and commercial mate, we found that the saponin pattern in *I. aquifolium* is less complicated than in *I. paraguariensis*, as shown in Table S3 (available in Supplementary Materials). Therefore, we have tentatively designated 29 and 53 compound respectively, as triterpene glycosides or glycoside esters. 

Qualitatively, the profiles were quite different: 16 compounds were found in both species, while 50 were unique to one or the second. In *I. paraguariensis*, one compound (911, 12.54 min; only in mate), tentatively assigned as matesaponin 1, was the dominant one, while another compound (911, 13.98 min; in both species) appeared to be the main saponin in *I. aquifolium*. However, numerous already-recognized saponins in the *Ilex* genus match the MS fragmentation of the last saponin, for example, ilexsaponin B2, latifoloside A, latifoloside B, or latifoloside D; thus, we cannot speculate on dereplication.

Considering the compounds classified as the same in both holly and mate, only six of sixteen had quite small differences below 10 RA% between species (911, 6.81 min; 825, 7.74 min; 927, 7.97 min; 911, 9.25 min; 1073, 9.53 min; 895, 14.68 min); these compounds, moreover, were also of generally small intensity. Four compounds differed between species in the range of 10–25 RA% (927, 8.30 min; 1219.61, 9.15 min probably matesaponin 4; 1057, 10.85 min; 1057, 12.15 min), while six others had a difference greater than 25RA%.

Taking into account the MS fragmentation of saponins, the following three types of triterpene aglycones were found based on MS/MS-resulting ions with *m*/*z* 455 for [C_30_H_48_O_3_−H]^−^ (an equivalent of monohydroxylated triterpene acid, such as oleanolic or ursolic acid), 469 for [C_30_H_46_O_4_−H]^−^ (an equivalent of didehydrogenated dihydroxylated triterpene acid) and 471 for [C_30_H_48_O_4_−H]^−^ (an equivalent of dihydroxylated triterpene acid) in both species.

### 2.4. Fatty Acid Profile in Ilex Leaves

Analysis of fatty acid methyl esters (FAMEs) from European *Ilex* cultivars revealed the presence of 17 compounds, and seven of the main FAMEs are summarized in Table 3 (in addition, Table S4 showing all compounds is available in Supplementary Data). It was found that the fatty acid profiles of the Argentine and European varieties were similar to one another. The main compounds identified in the European varieties were palmitic acid (12.05–15.03 mg g^−1^), linoleic acid (LA) (5.52–10.40 mg g^−1^) and α-linolenic acid (ALA) (9.09–22.16 mg g^−1^). Regarding the European cultivars, greater profile similarity was observed between *I. aquifolium* Ferox Argentea and *I. meserveae* Blue Angel due to similar amounts of LA (5.84 and 5.63 mg g^−1^) and ALA (9.72 and 9.09 mg g^−1^) acids. However, the other cultivars, i.e., *I. aquifolium* Alaska, *I. aquifolium* Rubricaulis Aurea, *I. meserveae* Golden Girl, and *I. meserveae* Blue Boy, showed higher amounts of these acids, which were as follows: LA (8.40–10.08 mg g^−1^) and ALA (19.28–22.16 mg g^−1^). *I. paraguriensis* as a reference sample showed the highest amounts of palmitic (18.08 mg g^−1^) and oleic acid (9.03 mg g^−1^). Stearic and α-linolenic acids were identified at similar levels, namely 6.39 and 5.92 mg g^−1^ respectively. It was also observed that the level of linoleic acid was lower in the Argentinean cultivar compared to the European cultivars.

The fatty acid profile in *I. paraguriensis* has also been reported by other researchers. In a paper published by Souza et al. [6], the acid that was significantly prominent in terms of the amount in the leaves of *I. paraguariensis* was α-linolenic acid, and the second acid that was most prominent, but in a much lower amount, was palmitic acid. Compared to the above analyses, the levels of palmitic and LA acids were lower. In addition, in the work of Reis et al. [20] the fatty acid profile of *I. paraguariensis* was demonstrated, where the main acids were also palmitic acid and α-linolenic acid, but their amounts were similar. As in the previous study, Reis et al. also reported lower contents of stearic acid, oleic acid, and linoleic acid. Chóez-Guaranda et al. [21], in their study on the oxidative activity of fractions of *I. guayusa* leaves, proved the presence of the following fatty acids in the hexane fraction: palmitic acid, oleic acid, and stearic acid.

Similar fatty acid relationships have been identified in medicinal plants in previous studies. Guil-Guerrero [22], in a study on *Plantago major*, determined the highest amount of α-linolenic acid (40.04%), followed by palmitic acid (16.59%) and linoleic acid (13.77%). The relationships among fatty acid profiles in medicinal plants were also recorded by Rutto [23], who explored the dietary value of raw and processed *Urtica dioica*. Stinging nettle showed the highest α-linolenic acid content (49.55%). The opposite situation to the previous case was also observed here, since the second highest acid content was attributed to linoleic acid (23.30%), followed by palmitic acid (17.06%). However, in the fatty acid profile of *Melissa officinalis* leaves, linoleic acid was found to be the dominant acid (74.08%), while palmitic acid was next (15.77%) [24]. In the leaves of *Salvia officinalis*, Taarit [25] identified those with 18 carbons in the molecule as the main fatty acids. The acid content decreases with the decrease in the number of double bonds in the acid (or with the decrease in the degree of unsaturation) and is as follows: α-linolenic acid (45.80%), linoleic acid (14.00%) and oleic acid (9.41%).

The polyunsaturated fatty acid/saturated fatty acid (PUFA/SFA) ratio is used when estimating the effect of diet on the cardiovascular system. PUFAs are assumed to lower serum low-density cholesterol (LDL), as opposed to SFAs, which contribute to high serum cholesterol [26]. It follows that the higher the PUFA/SFA ratio, the more positive the effect on the cardiovascular system. The most beneficial plants were *I. meserveae* Golden Girl (1.90), *I. aquifolium* Alaska (1.96) and *I. aquifolium* Rubricaulis Aurea (1.70), showing the highest indices; thus, compared to *I. paraguariensis* (0.57), they can be considered more beneficial to the circulatory system.

However, the PUFA/SFA ratio is too general and does not allow for an assessment of atherogenicity. Therefore, an atherogenicity index (IA) is used, which reveals the ratio of the sum of SFAs and the sum of unsaturated acids (UFAs). SFAs (C12:0, C14:0, C16:0) promote lipid adhesion to the cells of the circulatory and immune systems. However, UFAs inhibit the accumulation of atherosclerotic plaques and influence the reduction of phospholipids, cholesterol, and esterified fatty acids, which is why they are considered anti-atherosclerotic [27]. According to this rule, products with lower IA may contribute to the reduction of total cholesterol and LDL cholesterol in plasma. The values of the IA index in different *Ilex* cultivars range from 0.42 to 0.77. The lowest and most similar values are shown by *I. aquifolium* Alaska (0.42), *I. aquifolium* Rubricaulis Aurea (0.43), *I. meserveae* Blue Boy (0.44), and *I. meserveae* Golden Girl (0.50). In contrast, the IA indices of *I. aquifolium* Ferox Argentea (0.69) and *I. meserveae* Blue Angel (0.62) are similar to that of the reference sample of *I. paraguariensis* (0.77). This suggests that European cultivars may have a more beneficial effect than *I. paraguariensis* in lowering plasma cholesterol levels. An indicator that shows the tendency to form thrombi in blood vessels is the thrombogenicity index (IT). It characterizes the relationship between FAs that are considered prothrombogenic (C12:0, C14:0 and C16:0) and antithrombogenic (n-3, n-6 and MUFA). Therefore, a diet containing products with a lower IT ratio is more beneficial for the cardiovascular system [28]. The index of thrombogenicity in *Ilex* samples oscillates in the range of 0.20–0.69. The most favorable IT values, and thus indicating the most positive effect on the cardiovascular system, are observed in four European cultivars: *I. aquifolium* Alaska (0.20), *I. aquifolium* Rubricaulis Aurea (0.23), *I. meserveae* Golden Girl (0.22), and *I. meserveae* Blue Boy (0.22). However, the index of one of the European varieties of *I. aquifolium* Ferox Argentea (0.62) is similar to that of *I. paraguariensis* (0.69). In summary, both of these indices (IA and IT) can be used as a means to assess the effect of the composition of Fas on the cardiovascular system. Products with lower IA and IT compositions are associated with a reduced risk of coronary heart disease and better nutritional value.

Another indicator that can better illustrate the effect of the composition of Fas on cardiovascular disease than PUFA/SFA is the hypocholesterolemic/hypercholesterolemic index (HH). It describes the relationship between hypocholesterolemic fatty acids (cis-C18:1 and PUFA) and hypercholesterolemic Fas (C12:0, C14:0 and C16:0). As the value of the h/H index increases, the beneficial effect on the circulatory system also increases [29]. Here, the highest values were also found in *I. aquifolium* (2.68), *I. meserveae* Golden Girl (2.63), *I. aquifolium* Rubricaulis Aurea (2.41) and *I. meserveae* Blue Boy (2.39). They were twice as high as those of the reference samples, *I. paraguariensis* (1.24) and *I. aquifolium* Ferox Argentea (1.17).

Unsaturated fatty acids show different weights in relation to the unsaturation index (UI). This reflects the complex proportions of FA, with different degrees of unsaturation compared to the total FA composition. This index can be used to evaluate the suitability of the products as alternative sources of high-quality PUFAs [30,31]. Regarding the Argentine *I. paraguariensis* (110.07), all the European varieties tested obtained higher UI indices. The variety most similar to the reference sample was *I. meserveae* Blue Angel (126.82). The highest UI values were obtained for *I. aquifolium* (174.73) and *I. meserveae* Golden Girl (175.87).

The results obtained indicate that European *Ilex* varieties show potentially positive effects on human health through lowering cholesterol levels, reducing thrombus formation, and reducing the risk of cardiovascular disease. 

### 2.5. Animal Model

#### 2.5.1. Feed Consumption, Body Weight and Liver Weight

**Figure 1:**
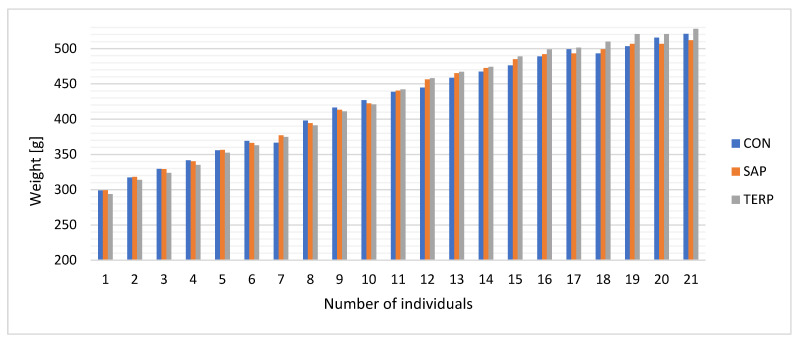
Weight changes after using saponins and terpenoids over a period of 8 weeks.

The initial body weight, the final body weight, and the food intake (Table 4) did not change between the groups. However, a tendency toward decreased body weight after the application of the terpenoid fraction was observed only at the final stage of the experiment. The dynamics of the changes in body weight during the supplementation period are presented in Figure 1. Feed intake in rats with metabolic syndrome affected liver weight, which was highest in the TERP group and lowest in SAP. These differences were not statistically confirmed. In another study, a hypercholesterolemic diet was shown to affect body weight [32]. Conversely, changes in adipose tissue stores may affect the expression of genes responsible for the synthesis of lipolytic hormones and trigger mechanisms related to cholesterol and free fatty acid levels. This has also been confirmed in our own studies. Obese Zucker rats had significantly higher body and organ weights and plasma levels of TNF-α, insulin, and leptin than lean animals [33]. Our own research suggests that these changes may be partially ameliorated by the use of saponins, especially with regard to body weight. 

#### 2.5.2. Hematological and Biochemical Parameters

The experimental group supplemented with terpenoids showed a significant increase (*p* < 0.05) in WBCs (Table 5) in the blood after 8 weeks compared to the control group. Similar changes were also found in the RBC (*p* < 0.01). In the group that received terpenoids, an increase in WBCs and RBCs was also observed, but no statistical differences were observed. The supplementation used also resulted in HGB and HCT (Table 5). No clear differences were found between the different red cell indices. Conversely, platelets reached the highest concentration in the TERP group, and were slightly lower for SAP, while PLT levels were lowest in the control group. Other studies indicate significantly higher plasma levels of RBC, WBC, monocytes and platelets, and leptin in obese rats [33].

The greatest changes in lipid parameters were found after terpenoid treatment (Table 6). There was a significant increase (*p* < 0.057) in TG concentration in the TERP group. Increases in the concentrations of total cholesterol, LDL and HDL fractions were also evident in the TERP group compared to the control group. However, these changes were not statistically significant. The use of saponins caused a slight decrease in the concentrations of cholesterol and LDL, and HDL. Supplementation with saponins caused a marked increase in TG concentrations compared to the control group. There was an increase in lipolysis in the TERP group, and the statistical differences compared to the SAP group were clear. The serum NEFA concentration was lowest in the SAP group. The administration of terpenoids or saponins to Zucker rats had no effect on AST activity.

Genetically obese Zucker (fa/fa) rats overexpress leptin in the stomach. Gastric leptin expression is regulated by feeding conditions in lean but not obese rats. This would indicate the impaired regulation of leptin expression in the stomachs of obese Zucker rats (as in adipose tissue), possibly related to the lack of the leptin receptor [34]. In a study in mice with an obesity-inducing diet, Yerba extract supplementation was shown to alleviate hyperglycemia and improve insulin sensitivity and plasma lipids [35]. Zucker rats receiving saponins and terpenoids had reduced serum insulin and glucose levels. This indicates their potential to reduce insulin resistance in obese Zucker rats. Our previous study showed that *I. aquifolium* and its terpenoids had an impact on serum lipid levels in an animal model of hyperlipidemia [32]. *I. aquifolium* improved insulin sensitivity and decreased blood concentrations of total cholesterol and LDL. Supplementation with terpenoids may be an effective means to reduce oxidative damage to lipids, as it decreases the concentration of MDA and leads to increased blood TAS. However, terpenoids also appeared to mitigate liver damage [32]. In general, a decrease in antioxidant capacity leads to endothelial dysfunction and is characterized by a decrease in NO bioavailability of vasodilator nitric oxide and an increase in endothelium-derived contractility factors, which lead to atherosclerosis [36]. In our study, age-related increases in terpenoids caused an increase in hyperlipidemia and total cholesterol levels, with a trend toward an increase in the LDL and HDL fractions. However, the most intense changes were observed in the TERP group.

#### 2.5.3. Oxidative Status

The mean values of the antioxidant status parameters are summarized in Table 7. Long-term administration of saponins or terpenoids to Zucker rats resulted in a significant increase (*p* < 0.01) in TAS compared to control rats. At the same time, the GR content decreased in the SAP group (*p* < 0.01). Terpenoid supplementation also caused a decrease in GR. The applied treatment did not have an effect on IL-6. IL-10 concentrations increased in both experimental groups, with the highest values reached in the TERP group. These changes were not statistically significant. Similarly, in the TERP and SAP groups, the increase in MCP-1 concentration was not statistically confirmed. Terpenoids did not have an effect on ROS values, while saponins caused a significant decrease (*p* < 0.01).

Previous studies have shown that metabolic syndrome characterized by obesity in Zucker rats decreased superoxide dismutase (SOD) activity in the plasma and heart, associated with cardiomyocyte hypertrophy [37]. Supplementation with saponins or terpenoids in our study increased TAS and decreased ROS, with no effect on blood NO concentration. As a consequence of obesity, the metabolic function of GPx was not affected to the same extent as the metabolic activity of SOD [37]. Both compounds tested caused a reduction in GR activity, but the effect of terpenoids was minor, and the effect of saponins was statistically significant (*p* < 0.01). Conversely, TNF- α and IL-6 are associated with a tendency to develop ischemia or atherosclerotic events [38]. In our study, there was no significant effect of saponins and terpenoids on blood concentrations of TNF- α and IL-6, and terpenoids caused an increase in IL-10. Interleukin 10 is considered an anti-inflammatory cytokine that inhibits the production of pro-inflammatory cytokines such as IL-2, IL-3, and TNF-α, for example. In an earlier study conducted on rats without metabolic syndrome (Wistar), TOS, GPx, and MDA were reduced after supplementation with terpenoids alone. No such effect was seen with water extracts of *Ilex* spp. [32].

#### 2.5.4. Fatty Acid Profile in Zucker Rat Livers

The chromatogram obtained for the rat liver differed from the chromatogram of fatty acids identified in the leaves of European holly species, primarily in terms of the presence of long-chain acids. Analyses showed that palmitic, elaidic, stearic, arachidonic, and linoleic acids were the most abundant in all test groups (Table 8). Supplementation with the terpenoid fraction and saponins had a significant effect on the change in fatty acid content in the rat liver. A decrease in elaidic acid was observed in the TERP group and an increase in the SAP group; its high content is not desirable due to a decrease in HDL cholesterol. For linoleic acid, no differences were observed between the CON and TERP groups, but a decrease was observed in the SAP group. With respect to arachidonic acid, an increase was observed in the TERP group and a decrease in the SAP group. An analogous trend was also observed for docosahexaenoic acid, which may be due to the conversion of α-linolenic acid, the content of which decreases significantly in both study groups. Comparing the content of saturated and unsaturated acids, SFA acids predominated in all groups. However, in the SAP group, the content of MUFA acids was higher compared to the TERP group. Conversely, the content of PUFA acids in the group fed with the saponin fraction was clearly lower. In total, PUFAω6 and PUFAω3 acids in the SAP group were also observed in a lower proportion than for the CON and TERP groups. 

The hepatic lipid profile in Zucker rats has also been investigated in other works. González-Torres et al. [39], in their study’s control group, showed the proportion of palmitic acid (16.31%), palmitoleic acid (20.40%), oleic acid (31.96%), arachidonic acid (9.78%) and docosahexanoic acid (3.45%) which together accounted for approximately 80% of the total lipid profile. Overall, the proportion of SFA and PUFA acids was similar, but the proportion of MUFA acids was twice as high. Conversely, Fiebig, who studied the effects of training on biological parameters in rats, found that in the group of untrained obese rats, the content of palmitic acid was 35.00%, with palmitoleic acid at 8.32%, oleic acid at 13%, and arachidonic acid at 6.78% [40]. In the study by Aguirre et al., feeding rats with pterostilbene caused an increase in total PUFAs, mainly through an increase in docosohexanoic acid and linoleic and arachidonic acids. The pterostilbene treatment decreased elaidic acid and the sum of MUFA [41].

#### 2.5.5. Transcription of Genes in the Aorta and Liver

**Figure 2:**
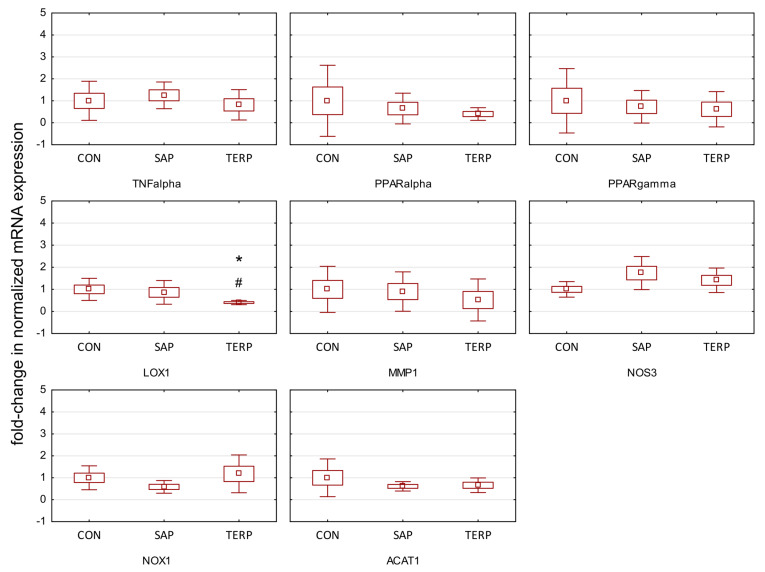
Expression of genes after treatment of saponin and terpenoid fraction in Zucker rat aorta. CON, control group; SAP, rats fed a standard diet with the addition of saponin fraction; TERP, rats fed a standard diet with the addition of terpenoid fraction. Values were normalized against Gapdh expression and presented as a fold-change in relation to gene expression in a control group. Specific comparisons: * *p* < 0.05 vs. CON; # *p* < 0.05 vs. SAP (ANOVA followed by the post hoc test). *TNFα*, tumor necrosis factor alpha; *PPARα*, peroxisome proliferator-activated receptor alpha; *PPARγ*, peroxisome proliferator-activated receptor gamma; *LOX1*, lectin-type oxidized LDL receptor; *MMP1*, matrix metalloproteinase-1; *NOS3*, nitric oxide synthase 3; *NOX1*, NADPH oxidase 1; *ACAT1*, acetyl-CoA acetyltransferase 1.

Increased TAS and decreased GR in the blood in both the SAP and TERP study groups indicate the plants’ antioxidant effects, and increased IL-10 in the blood in both study groups and decreased ROS in the SAP group indicate their anti-inflammatory effects. These findings are correlated with the increased expression of *LOX1* mRNA in the aorta (Figure 2). The expression of the receptor for oxidized LDL increases in the case of oxidative stress and inflammatory processes and is one of the most important markers of the increased oxidation of LDL particles with their subsequent incorporation into the arterial walls and formation of atherosclerotic plaques. Atherogenesis stimulating factors, *TNF-α*, oxLDL, and shear stress increase the expression of *LOX1* [42]. In our study, we found an increase in the expression of *NOS3* (eNOS) in the aorta in the SAP group (statistically significant) and in the TERP group (not statistically significant), arguing for increased endothelial nitric oxide synthase activity, leading to an increase in vascular endothelial NO, the main vasodilatory factor. Additionally correlated with these results was a decrease, although not statistically significant, in the expression of the metalloproteinase *MMP1* mRNA, evident in the TERP group. The reduction, although not statistically significant, in the expression of mRMA *NOX1* in the SAP group, indicates a reduction in oxidative stress in the aorta. There was also a reduction, although not statistically significant, in *ACAT1* in both groups; its increased expression was described in atherosclerotic plaques. Finally, although not statistically significant, *PPAR-α* and *PPAR-γ* mRNA expression can be interpreted as positive. Rats have different metabolic pathways than humans or rabbits. Many authors interpret the reduction in *PPAR* expression in rats as a positive indicator associated, for example, in the case of *PPAR-γ*, with reduced lipogenesis. Conversely, *PPAR-α* plays a pivotal role in controlling fatty acid oxidation. Synthetic *PPAR-α* agonists are currently used in the clinic as lipid-lowering and anti-atherosclerotic drugs. However, the potencies of these compounds at *PPAR-α* are lower than those of, e.g., monounsaturated FAE oleoylethanolamide and consequently do not significantly affect food intake [43].

**Figure 3:**
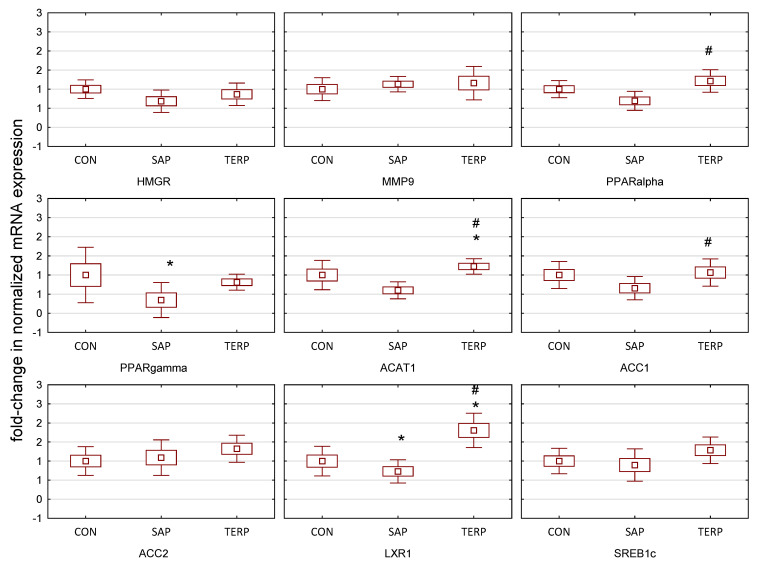
Expression of genes after treatment of saponin and terpenoid fraction in Zucker rat liver. CON, control group; SAP, rats fed a standard diet with the addition of saponin fraction; TERP, rats fed a standard diet with the addition of terpenoid fraction. Values were normalized against Gapdh expression and presented as a fold-change in relation to gene expression in a control group. Specific comparisons: * *p* < 0.05 vs. CON; # *p* < 0.05 vs. SAP (ANOVA followed by the post hoc test). *HMGR*, 3-hydroxy-3-methyl-glutaryl-coenzyme A reductase; *MMP*9, matrix metallopeptidase 9; *PPARalpha*, peroxisome proliferator-activated receptor alpha; *PPARγ*, peroxisome proliferator-activated receptor gamma*; ACAT*1, acetyl-CoA acetyltransferase 1; *ACC1*, acetyl-CoA carboxylase 1; *ACC*2, acetyl-CoA carboxylase 2; *LXR*1, liver X receptor; *SREB1c*, sterol regulatory element-binding protein 1.

In the livers (Figure 3) of the TERP group, expression of the transcription factor *LXR1*, which regulates cholesterol and fatty acid metabolism and reduces inflammatory processes, was increased [44]. LXR1 regulates fatty acid homeostasis through, among others, *SREBP-1c*, and this was increased, although not statistically significantly, in the TERP group. Conversely, mRNA expression of *HMGR*, a gene encoding a key enzyme in the endogenous synthesis of cholesterol in the liver, decreased in the SAP group, although not statistically significantly (statins also act in this mechanism, although they have pleiotropic effects, also positively influencing many other functions). In the SAP group, there was a decrease in *PPAR-α* and *PPAR-γ* mRNA expression. The interpretation of these changes is analogous to that for the aorta. The expression of *ACAT1* in the liver, responsible for the formation of cholesterol esters, decreased, but not statistically significantly, relative to the control group in the SAP group.

#### 2.5.6. Histopathological Examination

**Figure 4:**
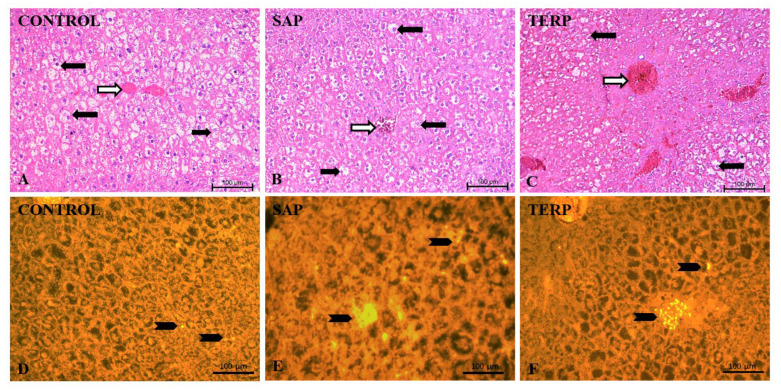
Representative pictures of rat liver stained with hematoxylin and eosin ((**A**–**C**) upper panel) and PMP70 immunohistochemical staining for the 70 kDa peroxisomal membrane protein marker PMP70 ((**D**–**F**) yellow signals in lower panel; solid arrowhead = positive signals detecting the presence of the peroxisomes in controls and experimental groups). Note the degree of steatosis—different amounts and sizes of the fat droplets in the hepatocytes (solid arrows = hepatocytes; open arrows = central vein) in pictures stained with hematoxylin and eosin. Mag. 200×. Scale bar 100 µm (**A**–**F**).

In the CON group (Figure 4A), histological analysis revealed deep liver steatosis in examined rats. The structure of the organ was blurred, and the portal bile spaces were invisible. The blood vessels were moderately filled with blood. There were cells of the white blood cell system within the larger blood vessels and in the periosteal spaces. Numerous apoptotic corpuscles were found in the periosteal spaces. There were no features associated with the severity of fibrinogenesis processes or fibroblast proliferation. Many hepatocytes showed double cell nuclei, which indicates an attempt to compensate for the processes associated with cell damage. The main change observed in the hepatocytes was the presence of accumulated lipids, which occupied most of the cell. These processes were observed most strongly in the peripheral areas of the lobules, while, in the area of the central vein, this process was much weaker and the hepatocytes contained a greater amount of cytoplasm.

Changes within the SAP group (Figure 4B) left a picture of fatty liver, but of lower intensity. Less blood vessels were filled with blood, and less swelling of the organ was observed. At the same time, the presence of portal bile spaces was observed between the hepatocytes. In the subcapsular areas, increased fibroblast activity was observed. Blood vessels were accompanied by leukocytic infiltrates.

In the TERP group (Figure 4C), the presence of a reduced amount of lipids was observed. No dividing cells or blood steatosis were found. The size of the hepatocytes decreased compared to that of the control group. In the cytoplasm, small lipid droplets were observed. 

In both experimental groups (Figure 4E,F), an increase in the number of peroxisomes was observed compared to the CON group (Figure 4D). Usually, this increase is not observed in the entire lobuli, but as numerous spots in some neighboring cells (Figure 4E,D).

## 3. Materials and Methods

### 3.1. Plant Material

Fresh plant material in the form of leaves of *I. aquifolium* and *I. meserveae* was obtained from the University of Life Sciences’ own cultivation (Vegetable and Ornamental Plant Research and Education Station, Psary, Poland). The holly shrubs were grown at the Department of Horticulture in the ornamental plant collection. After harvesting, the cleaned leaves were freeze-dried for 24 h (Lyovac GT 2 apparatus). The lyophilized leaves were crushed to obtain homogeneous material. The reference material was dried leaves of *I. paraguariensis*, purchased from a local distributor.

### 3.2. Solid-Phase Micro Extraction (SPME) Analysis

Fresh 1 g of material was ground in mortar, transferred to a headspace vial, and stored in a water bath at 40 °C for 15 min. Additionally, 2.5 µg of 2-undecanone was added as an internal standard. The volatile compound fraction was analyzed using a 2 cm fiber (DVB/CAR/PDMS, Supelco, Bellefonte, PA, USA); 15 min exposure and analyte desorption at 220 °C for 3 min. Analysis was performed on a Varian CP-3800/Saturn 2000 (Varian, Walnut Creek, CA, USA) equipped with a Zebron ZB-5 MSI column (30 m, 0.25 mm, 0.25 µm; Phenomenex, Torrance, CA, USA). The instrument was operated under the following conditions: injector 220 °C, gas flow as 1 mL · min^−1^ with a split ratio of 20. The program was set as follows: 40 °C for 3 min, a rate of 5.0 °C × min^−1^ from 40 to 110 °C and a rate of 20 °C × min^−1^ from 110 to 270 °C.

### 3.3. Isolation and Analysis of Terpenoid Fraction

The fractional analysis of natural compounds from *Ilex* leaves was performed using a gas chromatograph coupled to a mass spectrometer (Shimadzu GC-MS QP 2020 single-quadrupole gas chromatograph mass spectrometer, Shimadzu, Kyoto, Japan). Analyses were performed in triplicate.

To isolate the terpenoid fraction, 100 mg of *Ilex* leaves was weighed and poured into 5 mL of dichloromethane. An internal standard of 0.5 mg of cholesterol was also added to the sample. The sample was centrifuged on a benchtop centrifuge at 14,000× *g* rpm for 2 min. The supernatant was collected and washed twice with 4 mL of distilled water, which was then removed. The organic fraction was filtered through a celite filter, transferred to a round bottom flask, and evaporated to dryness on a vacuum evaporator under reduced pressure. Then, 400 µL of pyridine and 50 µL of N,O-Bis (trimethylsilyl)trifluoroacetamide (BSTFA) were added, and the mixture was transferred to a vial. The derivatization process was carried out in GC-MS whisks on a heating panel at 70 °C for 20 min. The prepared samples were stored at −18 °C until GC-MS analyzes were performed. Analyses were performed in triplicate.

Separation of the compounds that occur in the terpenoid fraction was achieved using a Zebron ZB-5 capillary column (30 m, 0.25 mm, 0.25 µm; Phenomenex, Torrance, CA, USA). GC-MS analysis was performed according to the following parameters. Scans were performed from 40 to 600 *m*/*z* using electron impact ionization (EI) at 70 eV, in a mode of 10 scans × s^−1^. The carrier gas used was helium, with a flow rate of 23 mL × min^−1^, with a split ratio of 1:20. The program setup for the analysis was as follows: 180 °C for 1 min, a rate of 5.0 °C × min^−1^ from 180 to 300 °C for 15 min. The injector was maintained at 280 °C.

### 3.4. Isolation and Analysis of Saponins Fraction

Extraction of the saponin fraction was performed using a modified methodology developed by Włodarczyk et al. [45]. Lyophilized leaves of *I. aquifolium* and *I. paraguariensis* (60 g) were macerated for 24 h at room temperature using 600 mL of cold 70% methanol (performed twice). The next step was to combine the extracts, which was filtrated, and a solution of 30 g of lead (II) acetate trihydrate in 70% methanol was added to precipitate the polar components such as phenols, peptides, and polysaccharides. The extract was centrifuged for 15 min at 3000 rpm. The residue containing the precipitated ballast was removed. Residual lead ions in the saponin fraction were removed with the addition of diluted sulfuric acid. The supernatant was diluted with distilled water to a concentration of 40% methanol and then centrifuged. Subsequently, the supernatant was applied to a 20 g acetadecyl SPE column (J.T. Baker), and the saponin-rich fraction was recovered with a minimal volume of pure methanol. The resulting fraction was concentrated on a vacuum evaporator, and the residue was lyophilized.

A UHPLC Ultimate 3000 instrument (Thermo Fisher Sci., Waltham, MA, USA) combined with an ESI-qTOF Compact detector (Bruker Daltonics, Bremen, Germany) was used to analyze the components of the saponin fraction. The prepared extract was diluted in acetonitrile/water (1:1 *V*/*V*) to a final concentration of 20 µg mL, with an injection volume of 5 µL. Compound separation was achieved on a Kinetex column (C-18, 150 mm × 2.1 mm, 2.6 μm; Phenomenex) at a flow rate of 0.3 mL × min^−1^, at 30 °C. Two phases, A (water) and B (acetonitrile), both with 0.1% formic acid, were used for the gradient: 0–1 min (from 2 to 30%B), 1–30 min (to 60% B), 31–31.5 min (to 100% B), 31.5–35.5 min (100% B). The detector was operated in negative mode, and the main parameters of the instrument were the following: scan range 50–2200 *m*/*z*, temperature 200 °C, nebulizer pressure 1.5 bar, capillary voltage 2.2 kV, dry gas-nitrogen 7.0 L × min^−1^, collision energy 10 eV vs. 30 eV. The results were analyzed using Data Analysis software (Bruker Daltonics).

### 3.5. Isolation and Analysis of Fatty Acids in Leaves and Rat Liver

The Folch method was used to extract lipids from *Ilex* leaves and rat liver samples [46]. Samples of crushed leaves and freeze-dried liver in the amounts of 0.5 and 0.1 g, respectively, were poured over 10 mL of the solvent mixture chloroform:methanol (in ratio 4:1) and heptadecanoic acid as an internal standard. Samples were allowed to stand for 1 h under a cover at 25 °C (the operation was performed twice), and then the organic phase was filtered and transferred to a round-bottom flask to evaporate the solvents to dryness under reduced pressure on a rotary evaporator (Heidolph-VAP Core). The contents of the flask were hydrolyzed using 2 mL KOH/MeOH, and the entire flask was heated on a heating panel at 70 °C for 5 min. Then, 1.5 mL of 14% (*V*/*V*) BF_3_/MeOH was added as a catalyst for the fatty acid esterification reaction. After the extract was cooled, 1 mL NaCl and 4 mL hexane were added to the flask for phase separation. The mixture was shaken for 5 min by hand, and then the hexane fraction was collected. The organic fraction that already contained methyl acid esters was dried on anhydrous magnesium sulphate, filtered on a silica celite filter and transferred to round bottom flasks to concentrate the sample (to 1 mL) on a rotary evaporator. The thus-prepared sample was then transferred to a GC-MS vial and stored at −18 °C until GC-MS analysis.

Separation of fatty acid methyl esters isolated from *Ilex* leaves and rat liver was achieved on a Zebron ZB-FAME capillary column (60 m, 0.20 mm, 0.20 µm; Phenomenex, Torrance, CA, USA). The GC-MS apparatus was operated under the conditions listed below: scanning was set from 40 to 400 *m*/*z* in electron ionization (EI) at 70 °C, the carrier gas type was helium with a flow rate of 0.98 mL min^−1^ at ratio 1:20, the temperature program was as follows: 80 °C for 2 min; rate of 3.0 °C × min^−1^ from 80 °C to 180 °C; rate of 8 °C × min^−1^ from 180 °C to 240 °C for 4 min. The injector was maintained at 280 °C.

### 3.6. Analysis of GC-MS Data

The identification of all obtained components on GC-MS was performed by two independent methods: (1) comparison of the calculated retention indices (RI) by the retention index calculator (against n-alkanes or saturated fatty acids authentic standards) with the RI contained in the NIST 20 database or Lipids Library 1.0; (2) comparison of the obtained spectra with the NIST 20 databases. During data analysis, the following programs were used: AMDIS (v. 2.73) and GCMS Solution (v. 4.20).

### 3.7. Animal and Experimental Design

Six-week-old Zucker (Zucker fatty rats fa/fa) male rats (n = 24) were purchased (Charles River, Madison, WI, USA) and fed a standard food diet for two weeks to acclimate them to their new environment. Animals were housed in pairs under controlled conditions (22 ± 1.6 °C were maintained on a 12 h light:12 h darkness schedule with standard laboratory diet and water available ad libitum). At eight weeks of age, 24 Wistar rats were randomly assigned to 3 groups (n = 8 animals per group): rats fed standard feed (CON group), rats receiving 10 mg/kg body weight of the saponin fraction extracted from *I. aquifolium* (SAP group), and rats receiving 10 mg/kg body weight of supplemented terpenoid fraction (TERP group). The extracts were administered individually to animals per os. The duration of the experiment was 8 weeks. Rats were fed commercial pellets (containing 16% protein, 2.5% fat, and 7.5% maximum fiber and other nutritional additives, according to the AOAC methods, (2005)). Food intake was monitored, and rats were weighed twice per week. After 8 weeks of feeding, the rats were sacrificed one at a time from each of the four groups and blood and tissue samples collected.

All procedures regarding Zucker rats were in agreement with the Local Ethics Committee (decision no. 09/2019/P1, Wrocław, Poland) and associated guidelines from European Communities Council Directive (no. 86/609/CEE) governing animal protection. The experimental design adopted was due to the limitations imposed by the 3Rs rule.

### 3.8. Blood Biochemical Analysis

Blood samples were collected around 10 a.m., 4 to 5 h after feeding (before euthanasia). Blood was collected in serum in sterile test tubes (Sarsted, Poland), anticoagulant tubes (EDTA-K3; Sarsted, Poland), EDTA-containing tubes (VT-100 STK, 0.1 mL of EDTA, 0.47 mL/L: 21 *w*/*v*; CML, Nemours, France) and in tubes filled with heparin sodium salt (Sarsted, Poland). Hematological parameters analysis was performed using the ABC Vet analyzer (Horiba ABX Diagnostics, Montpellier, Grabels, France) immediately after blood collection. Parameters such as red blood cell (RBC), white blood cell (WBC), platelets (PLT), hemoglobin (HGB), hematocrit (HCT), mean corpuscular volume (MCV), mean corpuscular hemoglobin (MCH), mean corpuscular hemoglobin concentration (MCHC), lymphocytes (LYM), monocytes (MON) and granulocytes (GRA) were recorded. Blood samples for serum or plasma were centrifuged at 3000× *g* for 10 min at room temperature (two hours from collection), and samples were frozen (−20 °C) until analysis.

Biochemical studies were performed using a Pentra 400 (Horiba ABX Diagnostics, France). The following parameters were estimated: nonesterified fatty acids (NEFA) by enzymatic method, Randox reagents (Crumlin, Dublin, Ireland); triglycerides (TG) and total cholesterol by enzymatic methods, HORIBA ABX reagents (Montpellier, France); high-density lipoprotein cholesterol (HDL) and low-density lipoprotein cholesterol (LDL) by colorimetric assay (Horiba ABX, Montpellier, France), glucose oxidase method (Horiba ABX, Montpellier, France); aspartate aminotransferase (AST), aspartate aminotransferase (AST) activity to kinetic method, HORIBA ABX reagents (Horiba ABX, Montpellier, France); total protein (TP) and albumin (Alb) via colorimetric method (Horiba ABX, Montpellier, France).

The following parameters of antioxidant status were also determined. Total antioxidant capacity (TAS) in serum by the colorimetric method based on ABTS (2,2-azine-di-[3-ethylbenzothiazoline sulfate]ta) with peroxidase, and glutathione reductase (GR) in whole blood by enzymatic method. Glutathione reductase catalyzes the reduction of glutathione (GSSG) in the presence of NADPH, which is oxidized to NADP+. The decrease in absorbance was measured at 340 nm. Rat Monocyte Chemotactic Protein 1 (MCP-1)—Immunoenzymatic test—sandwich ELISA principle using an antibody specific for rat MCP-1. Kit for the determination of reactive oxygen species (ROS) from MyBioSource Enzyme Immunoassay (San Diego, CA, USA), enzyme immunoassay based on ROS-ROS antigen interactions (immunosorbtion), and the HRP colorimetric detection system for the detection of ROS target antigens in samples. The ELISA kit is designed to detect native and non-recombinant ROS. Nitric oxide (NO) ready kit for the determination of nitric oxide from MyBioSource (San Diego, CA, USA). Colorimetric test based on the quantification of total NO2-/NO3- as a measure of the NO level. The reaction products can be measured with a colorimetric reading at 550 nm. These measurements were conducted using a Synergy fluorescence, luminescence, and absorbance reader from BioTek Instruments (Winooski, VT, USA).

### 3.9. Quantitative Real-Time PCR (RT-qPCR) Analysis

Liver and aorta fragments of 25–30 mg were homogenized in lysis buffer (PureLink RNA Mini Kit, Thermo Fisher Sci., Waltham, MA, USA) using ceramic spheres and FastPrep 24 homogenizer (MP Biomedicals, Irvine, CA, USA). Total RNA was isolated using phenol–chloroform extraction and was purified using the PureLink RNA Mini Kit (ThermoFisher Scientifics, Waltham, MA, USA). On-column digestion with Dnase PureLink™ Dnase Set (PureLink™ Dnase Set, Thermo-Fisher Scientific) was applied to prevent contamination with genomic DNA. RNA purity and concentration were determined using NanoDrop 2000 (ThermoFisher Scientifics). RNA aliquots (1000 ng) were reverse transcribed using iScript™ cDNA Synthesis Kit (BioRad, Hercules, CA, USA) following the manufacturer’s instructions. Quantitative PCR (qPCR) was performed on the CFX96 Real-Time PCR system (BioRad) using SsoFast EvaGreen^®^ Supermix (BioRad) and specific primers listed in Table 9, synthesized by Genomed (Warsaw, Poland). RT-qPCR conformed to the Minimum Information for Publication of Quantitative Real-Time PCR Experiments (MIQE) guidelines [47]. The gene expressions in all groups were compared with control [48,49]. The specificity of the primers was tested by melting curve analysis and in high-resolution agarose electrophoresis (Lonza, Basel, Switzerland) with SYBR Green detection (Lonza). The qPCR cycling conditions were as follows: 30 s activation at 95 °C, 5 s denaturation at 95 °C, annealing/extension for 5 s at 61 °C, 40 cycles, followed by melting step (60–95 °C). 

Technical replicates were averaged, and the geometric mean of all Cq values in all samples was calculated and subtracted from the individual sample Cq, yielding ΔCq. Then, ΔCq values were linearized by 2^ΔCq^ conversion, followed by a normalization to *GADPH* expression. The obtained normalized relative quantity (NRQ) [50] was subjected to statistical analysis.

### 3.10. Histopathological Examination of Tissues

Samples were fixed in a 4% buffered formalin solution with a pH of 7.2–7.4 for 24 h. Then, the material was rinsed in running water for 24 h, dehydrated in a series of alcohols, and embedded in paraffin. Slides 7 μm thick were routinely stained with hematoxylin and eosin.

Slides after deparaffinization in xylene were rehydrated, permeabilized with 0.1% Triton™ X-100 for 15 min, and blocked with 2% BSA for 1 h at room temperature. The cells were labeled with PMP70 Rabbit Polyclonal Antibody (Invitrogen, Waltham, MA, USA) at 5 µg/mL in 0.1% BSA, incubated at 4 °C overnight and then labeled with Goat anti-Rabbit IgG (H + L), Superclonal™ Recombinant Secondary Antibody, Alexa Fluor 488 (Invitrogen, Paisley, UK). The material was analyzed on a Zeiss Axio Observer D1 inverted phase contrast fluorescence microscope.

### 3.11. Statistical Analysis

Statistical analyses were performed with Statistica version 13.3 (StatSoft, Kraków, Poland). In vivo samples were analyzed in duplicate; the values for each group represent the mean of eight animals (n = 8). All results were expressed as means and standard deviation, and all variables were normalized using the Shapiro–Wilk test. The data obtained were subjected to analysis of variance (ANOVA) using a linear model procedure. The significance of the differences between the test results was determined by the NIR test. The significance level was established for *p* < 0.05 to evaluate the differences between the studied groups.

## 4. Conclusions

This is the first paper to describe the beneficial effects of terpenoid and saponin extracts of *I. aquifolium* on lipid metabolism in the Zucker rat model. The composition of the volatile fractions of the cultivars was also investigated, where the predominant constituents were the terpenoid p-cymene, α-phellandrene, and the six- and seven-carbon aldehydes and alcohols. The total volatile compound content was 81.91 µg g^−1^ for the *aquifolium* cultivar and 93.6 mg g^−1^ for *paraguariensis*. The terpene fractions of *I. paraguariensis* and European cultivars were characterized by a similar profile of triterpenes (both amyrin isometries), alcohols, and terpenoid acids. The Alaska cultivar was characterized by the highest content (30.45 mg g^−1^). The saponin fractions of the *I. aquifolium* species were similar to those of *I. paraguariensis*. The dominant ones in the LC-MS image were ilexsaponins and matesaponins in the European species. None of the fractions tested changed the hematological parameters in Zucker rats. We confirmed the effect of the used supplementation on carbohydrate and lipid metabolism. SAP and TERP decreased glucose and insulin, which may indicate mitigation of insulin resistance in a metabolic model. Increased TAS and decreased GR in the blood in both the SAP and TERP study groups indicate their antioxidant effects, and increased IL-10 in blood in both study groups and decreased ROS in the SAP group indicate their anti-inflammatory effects. The tendency toward reduced body weight was attributed to the use of the saponin fraction only. Only the terpenoid fractions enhanced the effect of lipolysis and increased the blood concentrations of HDL, LDL, and TG. 

Administration of saponins caused decreased (*p* < 0.05) expression of *PPAR-γ* mRNA in comparison to the control group and decreased *PPAR-α* in comparison to the SAP group. Terpenoids significantly reduced (*p* < 0.05) LOX1 in the SAP group, indicating a reduction in oxidative stress in the aorta. The expression of *ACAT1* in the liver, responsible for the formation of cholesterol esters, decreased statistically significantly relative to the control group, and it was correlated with blood lipid parameters. Furthermore, improved hepatic architecture was observed in both the SAP and TERP groups, with a reduction in lipids in hepatocytes associated with a higher number of peroxisomes responsible for lipid turnover. According to previous observations, TERP had a much more intensive influence on liver lipolysis than SAP. Finally, it could be concluded that saponin and terpenoid extracts of European *Ilex* varieties could modulate lipid metabolism in the Zucker rat model and finally hepatic steatosis.

## Figures and Tables

**Table 1:** Aroma profile of Argentine *I. paraguariensis* and European *I. aquifolium* and *I. meserveae*.

Compound	RI Exp. ^1^	RI Lit. ^2^	*I. paraguariensis*	*I. aquifolium*			*I. meserveae*		
				Alaska	Ferox Argentea	Rubricaulis Aurea	Blue Angel	Blue Boy	Golden Girl
			Concentration (µg g^−1^) d.w						
Hexanal	802	800	13.80 ^3a^	- ^4^	-	-	1.52 ^c^	1.18 ^c^	2.45 ^b^
2-Methyl-2-pentenal	838	837	11.77	-	-	-	-	-	-
(*E*)-2-Hexenal	860	854	1.77 ^a^	0.68 ^a^	-	-	12.28 ^b^	20.97 ^d^	14.61 ^c^
3-Hexen-1-ol	864	856	-	0.14 ^a^	-	-	11.85 ^d^	8.52 ^c^	6.34 ^b^
α-Pinene	938	937	0.23 ^a^	1.98 ^d^	2.77 ^e^	3.94 ^f^	1.32 ^c^	0.62 ^b^	0.51 ^ab^
6-Methyl-5-heptene-2-one	960	956	25.73	-	-	-	-	-	-
α-Phellandrene	989	986	-	4.71 ^d^	3.68 ^c^	5.44 ^d^	2.39 ^b^	1.05 ^a^	1.16 ^a^
(*E*,*E*)-2,4-Heptadienal	1012	1012	14.83	-	-	-	-	-	-
p-Cymene	1027	1025	2.65 ^a^	74.40 ^f^	58.21 ^e^	82.62 ^g^	32.75 ^d^	20.20 ^b^	22.51 ^c^
(*E*,*E*)-3,5-Octadien-2-one	1074	1068	22.82	-	-	-	-	-	-
Total			93.60	81.91	64.66	92.00	62.11	52.54	47.58

^1^ Experimental retention indices calculated against n-alkanes. ^2^ Retention indices according to the NIST20 database. ^3^ Values are mean; values followed by the same letter within a row are not significantly different (*p* > 0.05, Tukey’s test). ^4^ not detected.

**Table 2:** Profile of triterpenoids in *I. paraguariensis* and various varieties of *I. aquifolium* and *I. meserveae*.

Compound, TMS ^1^	RI Exp. ^2^	RI Lit. ^3^	*I. paraguariensis*	*I. aquifolium*			*I. meserveae*		
				Alaska	Ferox Argentea	Rubricaulis Aurea	Blue Angel	Blue Boy	Golden Girl
			Concentration (mg g^−1^) d.w						
β-Amyrin	3369	3353	1.44 ^4a^	1.21 ^b^	0.90 ^d^	0.49 ^e^	1.87 ^c^	1.95 ^c^	1.43 ^a^
α-Amyrin	3412	3406	4.28 ^a^	3.22 ^b^	1.87 ^d^	0.36 ^e^	3.07 ^b^	1.75 ^d^	2.18 ^c^
Lupeol	3429	3435	1.05 ^a^	2.18 ^e^	1.39 ^b^	0.42 ^c^	1.86 ^d^	1.35 ^b^	2.53 ^f^
Uvaol	3531	3540	2.21 ^a^	2.42 ^a^	1.03 ^c^	0.37 ^d^	0.99 ^c^	1.70 ^b^	2.48 ^a^
Betulinic acid	3579	3588	1.42 ^a^	0.98 ^b^	0.50 ^d^	0.76 ^c^	0.19 ^e^	0.42 ^d^	0.59 ^cd^
Oleanolic acid	3593	3591	2.03 ^a^	4.89 ^e^	3.45 ^c^	2.87 ^b^	4.08 ^d^	6.52 ^f^	4.31 ^d^
Ursolic acid	3664	3657	1.23 ^a^	15.55 ^f^	10.22 ^c^	7.29 ^b^	14.44 ^e^	16.11 ^g^	13.51 ^d^
Total			13.66	30.45	19.36	12.56	26.50	29.80	27.03

^1^ All compounds are TMS derivatives. ^2^ Experimental retention indices calculated against n-alkanes. ^3^ Retention indices according to the NIST20 database. ^4^ Values are mean; values followed by the same letter within a row are not significantly different (*p* > 0.05, Tukey’s test).

**Table 3:** Fatty acid profile and nutritional indices in leaves of *I. paraguariensis* and various varieties of *I. aquifolium* and *I. meserveae*.

Fatty Acid ^1^		RI Exp. ^2^	RI Lit. ^3^	*I. paraguariensis*	*I. aquifolium*			*I. meserveae*		
					Alaska	Ferox Argentea	Rubricaulis Aurea	Blue Angel	Blue Boy	Golden Girl
				Concentration (mg g^−1^) d.w						
Palmitic acid	C16:0	1599	1600	18.08 ^4a^	12.05 ^d^	15.03 ^b^	12.66 ^d^	14.03 ^bc^	13.04 ^cd^	12.51 ^d^
Stearic acid	C18:0	1801	1800	6.39 ^a^	2.37 ^c^	4.17 ^b^	2.50 ^c^	4.12 ^b^	2.56 ^c^	2.60 ^c^
Oleic acid	C18:1ω9	1817	1819	9.03 ^a^	3.60 ^e^	4.39 ^c^	3.92 ^d^	7.61 ^b^	3.41 ^e^	3.37 ^e^
Linoleic acid	C18:2ω6	1867	1874	4.23 ^a^	8.40 ^d^	5.84 ^b^	8.72 ^e^	5.63 ^b^	8.21 ^c^	10.08 ^f^
α-Linolenic acid	C18:3ω3	1933	1928	5.92 ^a^	21.31 ^d^	9.72 ^b^	19.28 ^c^	9.09 ^b^	19.61 ^c^	22.16 ^d^
Arachidonic acid	C20:4ω3	2118	2115	2.71 ^a^	0.71 ^c^	9.11 ^e^	0.54 ^cd^	1.84 ^b^	0.40 ^d^	0.58 ^cd^
Docosahexaenoic acid	C22:6ω3	2419	2416	1.67 ^a^	0.53 ^cd^	1.73 ^a^	0.66 ^c^	0.93 ^b^	0.45 ^d^	0.66 ^c^
		∑SFA		48.27 ^a^	30.38 ^g^	43.26 ^b^	32.85 ^e^	40.78 ^c^	33.26 ^d^	30.82 ^f^
		∑UFA		51.74 ^a^	69.64 ^g^	56.74 ^b^	67.15 ^e^	59.24 ^c^	66.75 ^d^	69.21 ^f^
		∑MUFA		25.31 ^a^	11.15 ^e^	29.78 ^c^	11.91 ^d^	23.41 ^b^	10.38 ^f^	9.45 ^g^
		∑PUFAω6		12.28 ^a^	17.37 ^e^	14.24 ^b^	17.61 ^f^	14.87 ^c^	17.22 ^d^	18.64 ^g^
		∑PUFAω3		14.16 ^a^	41.12 ^f^	12.72 ^b^	37.63 ^d^	20.96 ^c^	39.15 ^e^	41.12 ^f^
		IA ^5^		0.77 ^a^	0.42 ^c^	0.69 ^a^	0.43 ^c^	0.62 ^ab^	0.44 ^bc^	0.50 ^bc^
		IT ^6^		0.69 ^a^	0.20 ^c^	0.62 ^a^	0.23 ^c^	0.46 ^b^	0.22 ^c^	0.22 ^c^
		PUFA/SFA ^7^		0.57 ^a^	1.96 ^d^	0.63 ^a^	1.70 ^c^	0.91 ^b^	1.74 ^c^	1.90 ^d^
		h/H ^8^		1.24 ^a^	2.73 ^d^	1.17 ^a^	2.41 ^c^	1.72 ^b^	2.39 ^c^	2.66 ^d^
		UI ^9^		110.07 ^a^	174.73 ^d^	142.87 ^c^	169.17 ^d^	126.82 ^b^	168.16 ^d^	175.87 ^d^

^1^ All compounds are expressed as GC-MS percentage of methyl esters; ^2^ experimental retention indices calculated against saturated fatty acids; ^3^ retention indices according to the Lipids Library 1.0; ^4^ values are mean; values followed by the same letter within a row are not significantly different (*p* > 0.05, Tukey’s test); ^5^ IA, Index of Atherogenicity; ^6^ IT, Index of Thrombogenicity; ^7^ PUFA/SFA, polyunsaturated fatty acid/saturated fatty acid; ^8^ h/H, Hypocholesterolemic/Hypercholesterolemic Index; ^9^ UI, Unsaturation Index.

**Table 4:** Body weight, feed intake and liver weight.

	Group			*p* Value
	CON	SAP	TERP	
Initial body weight (g)	298.86 ± 65.89 ^1^	299.29 ± 15.24	293.71 ± 14.02	0.282
Final body weight (g)	521.00 ± 44.39	512 ± 51.91	528.14 ± 35.18	0.346
Feed intake (g/week)	123 ± 8.98	118 ± 6.62	132 ± 9.32	0.682
Liver weight (g)	23.10 ± 2.01	22.73 ± 4.19	24.06 ± 1.77	0.534

^1^ Values are mean ± SD.

**Table 5:** Hematological parameters in Zucker rats after treatment with saponin and terpenoid fractions.

	Group			*p* Value
	CON ^1^	SAP ^2^	TERP ^3^	
WBC ^5^ G/L	6.16 ± 1.02 ^4a^	8.66 ± 1.10	9.17 ± 2.19 ^b^	0.046
RBC ^6^ T/L	6.73 ± 1.04 ^a^	7.38 ± 0.75	7.47 ± 1.24 ^b^	0.006
HGB ^7^ mmol/L	7.37 ± 0.95	7.81 ± 0.76	7.80 ± 1.30	0.100
HCT ^8^ L/L	0.32 ± 0.05 ^a^	0.35 ± 0.03	0.35 ± 0.05 ^b^	0.010
MCV ^9^ fL	47.29 ± 0.76	46.71 ± 1.25	46.71 ± 1.38	0.296
MCH ^10^ fmoL	1.10 ± 0.06	1.06 ± 0.03	1.04 ± 0.03	0.402
MCHC ^11^ mmol/L	23.31 ± 1.45 ^a^	22.54 ± 0.64 ^a^	22.36 ± 0.69 ^b^	<0.001
PLT ^12^ G/L	431.00 ± 242.83	575.14 ± 253.74	674.00 ± 180.63	0.003

^1^ Control group; ^2^ rats fed a standard diet with the addition of saponin fraction; ^3^ rats fed a standard diet with the addition of terpenoid fraction; ^4^ values are mean ± SD; different letters within a row indicate significant differences between groups (*p* < 0.05; ANOVA followed by the post hoc test); ^5^ WBC, white blood cells; ^6^ RBC, red blood cells; ^7^ HGB, hemoglobin; ^8^ HCT, hematocrit; ^9^ MCV, mean corpuscular volume; ^10^ MCH, mean corpuscular hemoglobin; ^11^ MCHC, mean corpuscular hemoglobin concentration; ^12^ PLT, platelets.

**Table 6:** Mean values of lipid and biochemical parameters and activity of AST in blood Zucker rats.

	Group			*p* Value
	CON ^1^	SAP ^2^	TERP ^3^	
Glucose mmol/L	6.54 ± 0.62 ^4^	5.39 ± 1.59 ^4^	5.56 ± 1.85 ^4^	0.303
Insulin pg/mL	4015.80 ± 1714.38	3066.41 ± 1458.38	3078.84 ± 1134.45	0.394
Chol. ^5^ mmol/L	4.92 ± 0.34	4.63 ± 1.19	5.14 ± 0.93	0.574
HDL ^6^ mmol/L	2.11 ± 0.23	1.93 ± 0.52	2.13 ± 0.22	0.524
LDL ^7^ mmol/L	2.80 ± 0.29	2.71 ± 0.35	3.01 ± 0.27	0.426
TG ^8^ mmol/L	4.13 ± 0.82 ^a^	4.87 ± 1.94	6.09 ± 1.44 ^a^	0.057
NEFA ^9^ mmol/L	1.87 ± 0.42	1.63 ± 0.2 ^a^	2.15 ± 0.47 ^b^	0.062
AST ^10^ U/L	186.31 ± 74.71	152.29 ± 21.23	150.30 ± 27.16	0.304
TP ^11^ g/L	75.21 ± 4.61	73.99 ± 4.07	76.06 ± 5.26	0.711
Alb. ^12^ g/L	32.81 ± 2.62	34.70 ± 3.53	34.06 ± 1.34	0.419

^1^ Control group; ^2^ rats fed a standard diet with the addition of saponin fraction; ^3^ rats fed a standard diet with the addition of terpenoid fraction; ^4^ values are mean ± SD; different letters within a row indicate significant differences between groups (*p* < 0.05; ANOVA followed by the post hoc test); ^5^ Chol., cholesterol; ^6^ HDL, high-density lipoprotein; ^7^ LDL, low-density lipoprotein; ^8^ TG, triglycerides; ^9^ NEFA, non-esterified fatty acids; ^10^ AST, aspartate aminotransferase; ^11^ TP, total protein; ^12^ Alb., albumin.

**Table 7:** Effects of saponin and terpenoids on antioxidant and immunological parameters in Zucker rats.

	Group			*p* Value
	CON ^1^	SAP ^2^	TERP ^3^	
TAS ^5^ mmol/L	1.43 ± 0.29 ^4a^	1.82 ± 0.07 ^4b^	1.79 ± 0.04 ^4c^	0.001
GR ^6^ U/L	602.29 ± 373.21 ^a^	319.14 ± 120.27 ^b^	423.67 ± 211.92	0.030
IL-6 ^7^ pg/mL	1.22 ± 0.12	1.22 ± 0.26	1.21 ± 0.12	0.989
IL-10 ^8^ pg/mL	1.41 ± 1.84	1.95 ± 1.49	2.89 ± 1.40	0.237
MCP-1 ^9^ ng/mL	0.51 ± 0.27	0.72 ± 0.35	0.76 ± 0.16	0.214
ROS ^10^ U/mL	73.70 ± 3.14 ^a^	65.76 ± 4.77 ^b^	73.34 ± 6.48 ^b^	0.012
NO ^11^ µmol/L	0.19 ± 0.04	0.13 ± 0.09	0.17 ± 0.06	0.238

^1^ Control group; ^2^ rats fed a standard diet with the addition of saponin fraction; ^3^ rats fed a standard diet with the addition of terpenoid fraction; ^4^ values are mean ± SD; different letters within a row indicate significant differences between groups (*p* < 0.05; ANOVA followed by the post hoc test); ^5^ TAS, total antioxidant status; ^6^ GR, glutathione reductase; ^7^ IL-6, interleukin-6; ^8^ IL-10, interleukin-10; ^9^ MCP-1, blood monocyte chemotactic protein-1; ^10^ ROS, reactive oxygen species; ^11^ NO, nitric oxide.

**Table 8:** Fatty acid profile in Zucker rat liver.

Fatty Acid ^1^		RI Exp. ^2^	RI Lit. ^3^	Group		
				CON ^4^	SAP ^5^	TERP ^6^
				% of Total Hepatic Triacylglicerols		
Myristic acid	C14:0	1399	1400	2.36 ^7a^	2.56 ^b^	2.13 ^c^
Palmitic acid	C16:0	1598	1600	23.92 ^a^	25.54 ^b^	23.33 ^c^
Palmitoleic acid	C16:1ω7	1636	1632	9.56 ^a^	10.77 ^c^	9.02 ^b^
Stearic acid	C18:0	1800	1800	11.52 ^a^	12.05 ^c^	11.64 ^b^
Elaidic acid	C18:1ω9t	1821	1818	17.64 ^a^	21.44 ^c^	15.61 ^b^
Oleic acid	C18:1ω9	1818	1819	4.34 ^a^	4.62 ^c^	4.21 ^b^
Vaccenic acid	C18:1ω7	1829	1823	0.18 ^ab^	0.23 ^b^	0.15 ^a^
Linoleic acid	C18:2ω6	1866	1874	8.34 ^a^	6.75 ^b^	8.32 ^a^
γ-Linolenic acid	C18:3ω6	1899	1896	0.27 ^a^	0.20 ^b^	0.21 ^b^
α-Linolenic acid	C18:3ω3	1931	1928	1.79 ^a^	1.57 ^b^	1.40 ^c^
Gondoic acid	C20:1ω9	2018	2022	0.17 ^a^	0.12 ^a^	0.06 ^b^
Dihomo-γ-linolenic acid	C20:3ω6	2091	2096	1.76 ^a^	1.14 ^c^	1.68 ^b^
Arachidonic acid	C20:4ω6	2117	2115	9.01 ^a^	6.61 ^c^	11.71 ^b^
Eicosapentaenoic acid	C20:5ω3	2179	2181	1.22 ^a^	0.78 ^b^	1.23 ^a^
Docosapentaenoic acid	C22:5ω3	2359	2362	1.43 ^a^	1.06 ^c^	1.31 ^b^
Docosahexaenoic acid	C22:6ω3	2418	2416	6.44 ^a^	4.42 ^c^	8.03 ^b^
FA content of the liver (µg/mg)				215 ^a^	208 ^a^	172 ^b^
			∑SFA ^8^	37.81 ^a^	40.17 ^c^	37.07 ^b^
			∑MUFA ^9^	31.96 ^a^	37.26 ^c^	29.05 ^b^
			∑PUFA ^10^	30.22 ^a^	22.56 ^c^	33.88 ^b^
			∑PUFAω6	19.37 ^a^	14.71 ^c^	21.91 ^b^
			∑PUFAω3	10.85 ^a^	7.85 ^c^	11.97 ^b^

^1^ All compounds are expressed as GC-MS percentage of methyl esters; ^2^ experimental retention indices calculated against saturated fatty acids; ^3^ retention indices according to the Lipids Library 1.0. ^4^ control group; ^5^ rats fed a standard diet with the addition of saponin fraction; ^6^ rats fed a standard diet with the addition of terpenoid fraction; ^7^ values are mean; values followed by the same letter within a row are not significantly different (*p* > 0.05, Tukey’s test); ^8^ SFA, saturated fatty acid; ^9^ MUFA, monounsaturated fatty acids; ^10^ PUFA, polyunsaturated fatty acids.

**Table 9:** Primer sequences. *MMP1*, matrix metalloproteinase-1; *HMGR*, 3-hydroxy-3-methyl-glutaryl-coenzyme A reductase; *TNFα*, tumor necrosis factor alpha; *PPAR*α, peroxisome proliferator-activated receptor alpha; *PPAR*γ, peroxisome proliferator-activated receptor gamma; *ACAT*1, acetyl-CoA acetyltransferase 1; *ACC1*, acetyl-CoA carboxylase 1; *ACC*2, acetyl-CoA carboxylase 2; *LXR*1, liver X receptor; *SREB1c*, sterol regulatory element-binding protein 1; *LOX1*, lectin-type oxidized LDL receptor; *NOS3*, nitric oxide synthase 3; *NOX1*, NADPH oxidase 1.

Gene Symbol	Forward Primer	Reverse Primer
*Mmp1*	5′-CCACTAACATTCGAAAGGGTTT-3′	5′-GGTCCATCAAATGGGTTATTG-3′
*Hmgr*	5′-CTTGACGCTCTGGTGGAATG-3′	5′-GTTGGCAAGCACGGACATA-3′
*Tnfα*	5′-GCCCAGACCCTCACACTC-3′	5′-CCACTCCAGCTGCTCCTCT-3′
*Pparα*	5′-TCACACAATGCAATCCGTTT-3′	5′-GGCCTTGACCTTGTTCATGT-3′
*Pparγ*	5′-GAGATCCTCCTGTTGACCCAG-3′	5′-CCACAGAGCTGATTCCGAAGT-3′
*Acat1*	5′-CACAGAGCTGATTCCGAAGT-3′	5′-GAGCCATGCCTCTAGTACCT-3′
*Acc1*	5′-TCTATTCGGGGTGACTTTC-3′	5′-CAATCAGTCTGTCCAGCCA-3′
*Acc2*	5′-GGAACTCACGCAGTTGAGCAGG-3′	5′-CACATAAACCTCCAGGGACGCC-3′
*Lxr1*	5′-TGATGCTGAATTTGCTCTGC-3′	5′-GGCTCACCAGCTTCATTAGC-3′
*Sreb1c*	5′-TGCCCTAAGGGTCAAAACCA-3′	5′-TGGCGGGCACTACTCAGGAA-3′
*Lox1*	5′-TGATGCTGAATTTGCTCTGC-3′	5′-GGCTCACCAGCTTCATTAGC-3′
*Nos3*	5′-GGGCTCCCTCCTTCCGGCTGC-3′	5′-GGATCCCTGGAAAAGGCG-3′
*Nox1*	5′-TCTTGCTGGTTGACACTTGC-3′	5′-TATGGGAGTGGGAATCTTGG-3′

## Data Availability

The data presented in this study are available in Supplementary Materials.

## Supplementary Materials

The following supporting information can be downloaded at: https://www.mdpi.com/article/10.3390/molecules27113376/s1. Table S1: Aroma profile of Argentine *I. paraguariensis* and European *I. aquifolium* and *I. meserveae*. Table S2: Profile of triterpenoids in *I. paraguariensis* and various varieties of *I. aquifolium* and *I. meserveae*. Table S3: Saponins detected and provisionally identified in *Ilex paraguariensis* (Il.par) and *Ilex aquifolium* (Il.aq.) leaves extracts. Table S4: Fatty acid profile in leaves of *I. paraguariensis* and various varieties of *I. aquifolium* and *I. meserveae*.
