# Supplementary material for: Biochemical and Molecular Investigation of the Effect of Saponins and Terpenoids Derived from Leaves of Ilex aquifolium on Lipid Metabolism of Obese Zucker Rats
Source: Molecules. 2022 May 24;27(11):3376. doi: 10.3390/molecules27113376 (PMC9182309; doi:10.3390/molecules27113376)
Supplement: Supplementary file 1 [file molecules-27-03376-s001.zip › molecules-1722627-supplementary.pdf]

Table S1 Aroma profile of Argentine *I. paraguariensis* and European *I. aquifolium* and *I. meserveae*.

No.	RI exp.	RI lit.	Compound	*Ilex paraguariensis*	*Ilex aquifolium*			*Ilex meserveae*		
					Alaska	Ferox Agenta	Rubricaulis Aurea	Blue Angel	Blue Boy	Golden Girl
					Concentration (μg*g^{-1}) d.w					
1	753	754	(E)-2-Pentenal	2.48	-	-	-	-	-	-
2	764	765	1-Pentanol	1.38	-	-	-	-	-	-
3	766	767	(Z)-2-Penten-1-ol	1.34	-	-	-	-	-	-
4	780	782	3-Methyl-2-butenal	1.85	-	-	-	-	-	-
5	802	800	Hexanal	13.80	-	-	-	1.52	1.18	2.45
6	838	837	2-Methyl-2-pentenal	11.77	-	-	-	-	-	-
7	860	854	(E)-2-Hexenal	1.77	0.68	-	-	12.28	20.97	14.61
8	864	856	3-Hexen-1-ol	-	0.14	-	-	11.85	8.52	6.34
9	874	862	(E)-2-Hexen-1-ol	-	0.15	-	-	-	-	-
10	874	868	1-Hexanol	0.23	-	-	-	-	-	-
11	875	879	(Z)-4-Hexen-1-ol	-	-	-	-	1.68	2.50	3.48
12	876	890	5-hydroksy-Pentanal	0.82	-	-	-	-	-	-
13	893	891	2-Heptanone	2.50	-	-	-	-	-	-
14	903	901	Heptanal	4.48	-	0.05	0.01	0.17	0.13	0.06
15	918	911	(E,E)-2,4-Hexadienal	1.36	-	-	-	-	-	-
16	935	935	3-Hepten-2-one	0.28	-	-	-	-	-	-
17	938	937	α-Pinene	0.19	1.98	2.77	3.94	1.32	0.57	0.47
18	953	952	Camphene	-	0.08	0.08	0.14	0.10	0.02	0.02
19	960	956	2-Methyl-6-heptanone	1.88	-	-	-	-	-	-
20	965	962	Benzaldehyde	3.51	-	-	-	-	-	-
21	975	970	1-Heptanol	3.00	-	-	-	-	-	-
22	980	974	Sabinene	-	2.28	1.89	3.41	1.09	0.62	0.70
23	983	980	1-Octen-3-ol	0.69	-	0.02	-	-	-	-
24	989	986	6-Methyl-5-heptene-2-one	25.73	-	-	-	-	-	-
25	993	991	β-Myrcene	3.28	1.66	0.88	1.35	0.46	0.18	0.21
26	997	999	Furfuryl methyl sulfide	3.64	-	-	-	-	-	-

27	1002	1000	Decane	-	1.34	0.80	1.40	0.37	0.12	0.23
28	1003	1003	Octanal	5.88	-	-	-	-	-	-
29	1006	1005	α-Phellandrene	-	4.71	3.68	5.44	2.39	1.05	1.16
30	1012	1012	(E,E)-2,4-Heptadienal	14.83	-	-	-	-	-	-
31	1020	1017	α-Terpinene	-	0.43	0.30	0.49	0.15	-	0.08
32	1027	1025	p-Cymene	2.65	74.40	58.21	82.62	32.75	20.20	22.51
33	1032	1026	o-Cymene	2.41	2.35	1.02	1.70	0.55	0.10	0.25
34	1043	1031	(E)-Oct-3-en-2-one	2.09	-	-	-	-	-	-
35	1047	1032	Sylvestrene	0.37	-	-	-	-	-	-
36	1053	1037	β-Ocimene	0.28	-	-	-	-	-	-
37	1057	1054	Prenyl isobutyrate	2.31	-	-	-	-	-	-
38	1063	1060	γ-Terpinene	4.78	1.27	0.74	1.23	0.46	0.21	0.32
39	1067	1067	3,5,5-Trimethylcyclohex-3-en-1-ol	0.56	-	-	-	-	-	-
40	1074	1068	(E,E)-3,5-Octadien-2-one	22.82	-	-	-	-	-	-
41	1078	1071	1-Octanol	-	-	-	-	0.41	0.16	-
42	1090	1074	Linalool oxide	1.81	-	-	-	-	-	-
43	1093	1090	p-Cymenene	-	2.21	0.98	0.92	1.18	2.14	2.55
44	1095	1091	3,5-Octadien-2-one	5.17	-	-	-	-	-	-
45	1101	1099	Linalool	6.47	-	-	-	0.45	0.98	0.45
46	1106	1102	2-Nonen-1-ol	4.12	-	-	-	-	-	-
47	1109	1104	α-Thujone	-	0.37	0.29	1.16	0.83	0.62	0.80
48	1110	1108	Maltol	1.75	-	-	-	-	-	-
49	1122	1119	3-Thujanone	-	-	-	-	-	0.16	0.33
50	1138	1142	3-Nonen-2-one	2.54	-	-	-	-	-	-
51	1147	1143	(E)-Sabinol	0.93	4.01	2.69	0.27	-	6.39	3.03
52	1151	1145	Camphor	1.36	0.22	0.93	0.98	0.72	0.82	0.65
53	1163	1153	Citronellal	0.41	-	-	-	-	-	-
54	1176	1167	endo-Borneol	-	-	-	-	0.26	0.95	0.16
55	1183	1173	Isocamphopinone	-	-	-	-	0.08	0.10	0.08
56	1189	1182	Naphthalene	2.44	-	-	-	-	-	-
57	1195	1189	α-Terpineol	1.62	-	-	-	-	-	-

58	1197	1190	Methyl salicylate	2.28	-	-	-	-	-	-
59	1208	1196	Estragole	1.14	2.03	2.48	3.49	2.59	3.12	3.41
60	1229	1220	β-Cyclocitral	2.07	-	-	-	-	-	-
61	1245	1228	Citronellol	0.39	-	-	-	0.05	0.17	0.16
62	1251	1240	Neral	0.82	-	-	-	-	-	-
63	1265	1257	Linalool acetate	0.19	0.10	0.24	0.55	0.18	0.30	0.40
64	1270	1263	(E)-2-Decenal	1.53	-	-	-	-	-	-
65	1280	1270	Geranial	1.40	-	-	-	-	-	-
66	1363	1317	n-Butyric acid 2-ethylhexyl ester	2.76	0.10	0.09	0.09	0.14	0.16	0.05
67	1381	1380	Propanoic acid. 2-methyl-, 3-hydroxy-2,2,4-trimethylpentyl ester	4.59	0.09	0.10	0.13	0.02	-	-
68	1393	1386	β-Damascenone	0.80	-	-	-	-	-	-
69	1408	1408	6,10-Dimethyl-2-undecanone	0.91	-	-	-	-	-	-
70	1439	1426	α-Ionone	3.28	0.04	0.07	0.09	0.11	0.08	0.05
71	1459	1435	(Z)-Geranylacetone	4.40	-	-	-	-	-	-
72	1476	1454	Humulene		-	-	-	-	0.06	0.03
73	1497	1486	β-Ionone	3.94	-	-	-	0.03	-	-
74	1608	1600	Hexadecane	0.28	0.05	0.15	0.27	0.13	-	-
75	1611	1606	Geranyl isovalerate	0.41	0.03	0.07	0.32	0.05	0.74	0.28
76	1671	1649	Hedione	3.45	0.02	0.07	0.00	-	-	-
77	1702	1700	Heptadecane	1.44	-	-	-	0.14	0.55	0.26
78	1775	1755	α-hexyl-Cinnamaldehyde	0.37	0.07	0.39	0.42	-	-	-
79	1827	1818	Vetivenic acid	2.56	-			0.35	1.30	0.77
80	1855	1844	6,10,14-Trimethylpentadecan-2-one	0.32	0.20	1.05	0.81	-	-	-
81	1863	1863	Z-9-Hexadecen-1-ol	0.58	-	-	-	-	-	-

Table S2 Profile of triterpenoids in *I. paraguariensis* and various varieties of *I. aquifolium* and *I. meserveae*.

Compound, TMS[1]	RI Exp.[2]	RI Lit.[3]	*I. paraguariensis*	*I. aquifolium*			*I. meserveae*		
				Alaska	Ferox Argentea	Rubricaulis Aurea	Blue Angel	Blue Boy	Golden Girl
				Concentration (mg*g^{-1}) d.w					
α-Tocopherol	3221	3226	0.43	1.72	0.61	0.39	0.78	1.23	0.91
β-Sitosterol	3342	3348	0.86	1.53	1.44	0.83	1.34	1.28	0.97
β-Amyrin	3369	3353	1.44	1.21	0.90	0.49	1.87	1.95	1.43
Germanicol	3382	3385	0.05	0.19	0.08	0.06	0.11	0.11	0.16
α-Amyrin	3412	3406	4.28	3.22	1.87	0.36	3.07	1.75	2.18
Lupeol	3429	3435	1.05	2.18	1.39	0.42	1.86	1.35	2.53
Epilupeol	3420	3439	0.54	0.12	0.18	0.12	0.22	0.15	0.10
Unknown	3502	-	0.21	0.36	0.19	0.10	0.21	0.34	0.46
Uvaol	3531	3540	2.21	2.42	1.03	0.37	0.99	1.70	2.48
Betulinic acid	3579	3588	1.42	0.98	0.50	0.76	0.19	0.42	0.59
Oleanolic acid	3593	3591	2.03	4.89	3.45	2.87	4.08	6.52	4.31
Ursolic acid	3664	3657	1.23	15.55	10.22	7.29	14.44	16.11	13.51

[1] All compounds are TMS derivatives. [2] Experimental retention indices calculated against n-alkanes. [3] Retention indices according to the NIST20 database.

Table S3 Saponins detected and provisionally identified in *Ilex paraguariensis* (Il.par) and *Ilex aquifolium* (Il.aq.) leaves extracts.

No.	provisional name [pseudomolecular ion & retention time]	neutral formula	error [ppm]	RA [%] (Il.par.)	RA [%] (Il.aq.)	reported in [1]	MS mode fragments /30 eV/ (intensity%)	MS2 mode fragments /adjusted collision energy/	provisional identification based on *Ilex* saponins database and fragmentation pathway
**1**	1089, 6.29 min	$C_{53}H_{86}O_{23}$	6.2	—	1.64		1089.5420 (100) $[M-H]^-$	—	—
**2**	1089, 6.37 min	$C_{53}H_{86}O_{23}$	6.2	—	1.07		1089.5420 (100) $[M-H]^-$	—	—
**3**	911, 6.60 min	$C_{47}H_{76}O_{17}$	2.7	0.79	—		911.4985 (100) $[M-H]^-$	—	—
**4**	911, 6.81 min	$C_{47}H_{76}O_{17}$	4.7	1.35	2.29		911.4967 (100) $[M-H]^-$	—	—
**5**	1073, 7.07 min	$C_{53}H_{86}O_{22}$	4.3	1.10	—		1073.5492 (100) $[M-H]^-$	—	—
**6**	1089, 7.11 min	$C_{53}H_{86}O_{23}$	0.7	—	2.14		1089.5479 (100) $[M-H]^-$	—	—
**7**	927, 7.17 min	$C_{47}H_{76}O_{18}$	−0.8	2.76	—		927.4966 (100) $[M-H]^-$	—	—
**8**	1089, 7.22 min	$C_{53}H_{86}O_{23}$	6.3	—	0.82		1089.5419 (100) $[M-H]^-$	—	—
**9**	927, 7.41 min	$C_{47}H_{76}O_{18}$	6.8	0.85	—		927.4896 (100) $[M-H]^-$	—	—
**10**	911, 7.53 min	$C_{47}H_{76}O_{17}$	1.8	—	12.99		911.4993 (100) $[M-H]^-$	—	—
**11**	1073, 7.64 min	$C_{53}H_{86}O_{22}$	3.9	1.25	—	+	1073.5496 (100) $[M-H]^-$	—	—
**12**	825, 7.74 min	$C_{42}H_{66}O_{16}$	−0.3	7.23	13.15	+	825.4291 (100) $[M-H]^-$	/65.5 eV/<br>825.43 (100) $[M-H]^-$<br>663.37 (22) $[M-Hex-H]^-$<br>487.34 (63) $[AGL-H]^-$ =<br>$[M-Hex-HexA-H]^-$<br>455.32 (28) $[AGL-H_2O-H]^-$	ilexoside XLVI,<br>ilexoside XLVII,<br>ilexoside XXXIX
**13**	1073, 7.79 min	$C_{53}H_{86}O_{22}$	6.6	2.31	—		1073.5467 (100) $[M-H]^-$	—	—
**14**	927, 7.85 min	$C_{47}H_{76}O_{18}$	−0.4	0.87	—		927.4963 (100) $[M-H]^-$	—	—
**15**	1073, 7.92 min	$C_{53}H_{86}O_{22}$	5.4	2.84	—	+	1073.5480 (100) $[M-H]^-$	—	—
**16**	927, 7.97 min	$C_{47}H_{76}O_{18}$	2.6	0.84	9.76		927.4935 (100) $[M-H]^-$,<br>779.4592 (48)	—	—
**17**	1073, 8.09 min	$C_{53}H_{86}O_{22}$	1.8	17.19	44.27	+	1119.5567 (24) $[M+FA-H]^-$,<br>1073.5519 (37) $[M-H]^-$,<br>911.5010 (100) $[M-Hex-H]^-$,<br>809.4272 (10)	/83.9 eV/<br>911.50 (28) $[M-Hex-H]^-$,<br>765.44 (16) $[M-Hex-dxHex-H]^-$,<br>749.45 (100.0) $[M-Hex-Hex-H]^-$,<br>603.39 (19) $[M-Hex-Hex-dxHex-H]^-$,<br>471.35 (17) $[AGL-H]^-$ =<br>$[M-Hex-Hex-dxHex-Pen-H]^-$	latifoloside C,<br>kudinoside G,<br>latifoloside L,<br>latifoloside E
**18**	927, 8.15 min	$C_{47}H_{76}O_{18}$	8.2	3.60	—		927.4883 (100) $[M-H]^-$	—	—

No.	provisional name [pseudomolecular ion & retention time]	neutral formula	error [ppm]	RA [%] (Il.par.)	RA [%] (Il.aq.)	reported in [1]	MS mode fragments /30 eV/ (intensity%)	MS2 mode fragments /adjusted collision energy/	provisional identification based on *Ilex* saponins database and fragmentation pathway
**19**	927, 8.30 min	C47H76O18	0.2	7.72	27.22	+	927.4957 (65) [M−H]⁻, 765.4428 (100) [M−Hex−H]⁻	/72.2 eV/ 765.44 (100) [M−Hex−H]⁻, 603.40 (97) [M−Hex−Hex−H]⁻, 469.76 [AGL-H_2−H]⁻ = [M−Hex−Hex−Pen-3H]-	godoside D, ilekudinoside E, ilexoside II, ilexoside XV, ilexsaponin B3
**20**	1101, 8.52 min	C54H86O23	3.6	1.88	—		1101.5448 (39) [M−H]⁻, 939.4933 (100) [M−Hex−H]⁻	—	—
**21**	1381, 8.56 min	C65H106O31	4.4	11.17	—	+	1381.6590 (100) [M−H]⁻	/108.5 eV/ 895.50 (9) [M−Hex−Hex−Hex−H]⁻, 733.45 (82) [M−Hex−Hex−Hex−Hex−H]⁻, 587.40 (77) [M−Hex−Hex−Hex−Hex−dxHex−H]⁻, 455.35 (100) [AGL−H]⁻ = [M−Hex−Hex−Hex−Hex−dxHex−Pen−H]⁻	matesaponin 5
**22**	1131, 8.75 min	C55H88O24	4.8	5.64	—	+	1131.5539 (55) [M−H]⁻, 969.5026 (100) [M−Hex−H]⁻	—	—
**23**	927, 8.75 min	C47H76O18	−0.3	0.88	—		927.4962 (100) [M−H]⁻	—	—
**24**	1101, 8.91 min	C54H86O23	4.6	5.28	—	+	1101.5436 (43) [M−H]⁻, 939.4918 (100) [M−Hex−H]⁻	—	—
**25**	809, 9.10 min	C42H66O15	1.8	36.02	—		809.4314 (100) (M−H]⁻	/64.6 eV/ 809.43 (100) [M−H]⁻ 647.38 (46) [M−Hex−H]⁻ 471.34 (37) [AGL−H]⁻ = [M−Hex−HexA−H]⁻	ilekudinoside B, ilexoside XXXI, ilexoside XLVIII, ilexoside L
**26**	1219.61, 9.15 min	C59H96O26	5.1	57.97	37.27	+	1219.6120 (100) [M−H]⁻, 895.5041 (78) [M-(Hex+Hex)−H]⁻	/95.6 eV/ 733.46 (96) [M−Hex−Hex−Hex−H]⁻, 587.39 (66) [M−Hex−Hex−Hex−dxHex−H]⁻, 455.35 (100) [AGL−H]⁻ = [M−Hex−Hex−Hex−dxHex−Pen−H]⁻	matesaponin 4
**27**	911, 9.25 min	C47H76O17	0.3	5.37	10.50	+	911.5007 (100) [M−H]⁻	—	—
**28**	809, 9.29 min	C42H66O15	1.4	16.93	—		809.4317 (100) [M−H]⁻	/64.6 eV/ 809.43 (100) [M−H]⁻ 647.38 (36) [M−Hex−H]⁻	ilekudinoside B, ilexoside XXXI, ilexoside XLVIII, ilexoside L

No.	provisional name [pseudomolecular ion & retention time]	neutral formula	error [ppm]	RA [%] (Il.par.)	RA [%] (Il.aq.)	reported in [1]	MS mode fragments /30 eV/ (intensity%)	MS2 mode fragments /adjusted collision energy/	provisional identification based on *Ilex* saponins database and fragmentation pathway
								471.34 (22) [AGL−H]$^-$ = [M−Hex−HexA−H]$^-$	
**29**	1073, 9.53 min	C53H86O22	5.4	6.76	1.89	+	1073.5480 (44) [M−H]$^-$, 765.4423 (100) [M−Hex−dxHex−H]$^-$	—	—
**30**	1073, 9.64 min	C53H86O22	−2.1	—	2.29		1073.5560 (68) [M−H]$^-$, 911.5013 (100) [M−Hex−H]$^-$	—	—
**31**	1073, 9.90 min	C53H86O22	−2.8	1.61	—	+	1073.5568 (100) [M−H]$^-$	—	—
**32**	911, 10.04 min	C47H76O17	0.7	—	3.27		911.5003 (100) [M−H]$^-$	—	—
**33**	1277, 10.20 min	C62H98O28	5.5	3.81	—	+	1277.6102 (100) [M−H]$^-$	—	—
**34**	1247, 10.48 min	C60H96O27	4.1	9.43	—	+	1247.6015 (100) [M−H]$^-$	/97.8 eV/ 881.49 (14) [M−Ac−Hex−Hex−H]$^-$, 749.44 (49) [M−Ac−Hex−Hex−Pen−H]$^-$, 587.40 (100.0) [M−Ac−Hex−Hex−Pen−Hex−H]$^-$, 455.35 (36) [AGL−H]$^-$ = [M−Ac−Hex−Hex−Pen−Hex−Pen−H]$^-$	acetylated ilexoside X, acetylated ilexoside XX
**35**	1235, 10.57 min	C62H92O25	4.4	5.60	—		1235.5800 (100) [M−H]$^-$, 1073.5282 (18) [M−Hex−H]$^-$	—	—
**36**	1235, 10.70 min	C62H92O25	3.6	5.42	—		1235.5810 (100) [M−H]$^-$, 1073.5272 (18) [M−Hex−H]$^-$	—	—
**37**	1057, 10.85 min	C53H86O21	4.6	40.28	16.38	+	1057.5540 (100) [M−H]$^-$	/82.6 eV/ 733.4545 (100) [M−Hex−Hex−H]$^-$, 455.3508 (3.5) [AGL−H]$^-$ = [M−Hex−Hex−dxHex−Pen−H]$^-$	ilekudinoside A, matesaponin 2
**38**	927, 10.91 min	C47H76O18	1.5	—	17.71	+	927.4945 (100) [M−H]$^-$	/72.2 eV/ 765.45 (100) [M−Hex−H]$^-$ 487.33 (71) [M−Hex−dxHex−Pen−H]$^-$	kudinoside A, kudinoside F
**39**	927, 11.06 min	C47H76O18	1.0	—	3.21	+	927.4950 (100) [M−H]$^-$	—	—
**40**	1235, 11.09 min	C62H92O25	4.9	7.86	—		1235.5794 (100) [M−H]$^-$	—	—
**41**	1235, 11.09 min	C62H92O25	6.0	10.00	—		1235.5781 (100) [M−H]$^-$	—	—
**42**	1115, 11.14 min	C55H88O23	7.0	5.50	—	+	1115.5566 (100) [M−H]$^-$	—	—

No.	provisional name [pseudomolecular ion & retention time]	neutral formula	error [ppm]	RA [%] (Il.par.)	RA [%] (Il.aq.)	reported in [1]	MS mode fragments /30 eV/ (intensity%)	MS2 mode fragments /adjusted collision energy/	provisional identification based on *Ilex* saponins database and fragmentation pathway
**43**	1115, 11.28 min	C55H88O23	4.7	29.29	—	+	1115.5592 (100) [M−H]⁻	/87.2 eV/<br>749.45 (48) [M−Ac−Hex−Hex−H]⁻,<br>731.44 (79),<br>587.40 (100) [M−Ac−Hex−Hex−Hex−H]⁻,<br>455.35 (29) [AGL−H]⁻ =<br>[M−Ac−Hex−Hex−Hex−Pen−H]⁻	acetylated matesaponin 3
**44**	1057, 11.90 min	C53H86O21	3.7	44.95	10.25	+	1057.5550 (39) [M−H]⁻,<br>895.5047 (100) [M−Hex−H]⁻	/82.6 eV/<br>895.51 (39) [M−Hex−H]⁻,<br>733.45 (100) [M−Hex−Hex−H]⁻,<br>587.40 (60) [M−Hex−Hex−dxHex−H]⁻,<br>455.35 (73) [AGL−H]⁻ =<br>[M−Hex−Hex−dxHex−Pen−H]⁻	ilekudinoside A,<br>matesaponin 2
**45**	911, 12.05 min	C47H76O17	1.6	6.80	—		911.4995 (100) [M−H]⁻	—	—
**46**	1057, 12.15 min	C53H86O21	4.4	26.92	8.40	+	1057.5542 (49.5) [M−H]⁻,<br>895.5047 (100) [M−Hex−H]⁻	/82.6 eV/<br>895.50 (27) [M−Hex−H]⁻,<br>733.45 (100) [M−Hex−Hex−H]⁻,<br>587.40 (60) [M−Hex−Hex−dxHex−H]⁻,<br>455.35 (49) [AGL−H]⁻ =<br>[M−Hex−Hex−dxHex−Pen−H]⁻	ilekudinoside A,<br>matesaponin 2
**47**	793, 12.31 min	C42H66O16	2.2	12.22	—		793.4362 (100) [M−H]⁻	/63.6 eV/<br>793.4331 (100) [M−H]⁻,<br>631.3840 (76) [M−Hex−H]⁻,<br>569.3845 (97),<br>455.3523 (32) [AGL−H]⁻ =<br>[M−Hex−HexA−H]⁻	chikusetsusaponin IV a
**48**	911, 12.54 min	C47H76O17	2.1	100.00	—	+	911.4991 (76) [M−H]⁻,<br>749.4472 (100) [M−Hex−H]⁻	/70.9 eV/<br>793.43 (68),<br>587.40 (75) [M−Hex−Hex−H]⁻,<br>569.39 (100),<br>455.35 (28) [AGL−H]⁻ =<br>[M−Hex−Hex−Pen−H]⁻	matesaponin 1
**49**	911, 12.74 min	C47H76O17	3.4	14.06	—	+	911.4979 (96) [M−H]⁻,<br>749.4463 (100) [M−Hex−H]⁻	—	—
**50**	911, 12.94 min	C47H76O17	3.5	8.48	—	+	911.4978 (58) [M−H]⁻,<br>749.4461 (100) [M−Hex−H]⁻	—	—
**51**	1115, 13.34 min	C55H88O23	7.6	1.56	—	+	1115.5559 (100) [M−H]⁻	—	—

No.	provisional name [pseudomolecular ion & retention time]	neutral formula	error [ppm]	RA [%] (Il.par.)	RA [%] (Il.aq.)	reported in [1]	MS mode fragments /30 eV/ (intensity%)	MS2 mode fragments /adjusted collision energy/	provisional identification based on *Ilex* saponins database and fragmentation pathway
**52**	1085, 13.69 min	C54H86O22	4.8	3.57	—	+	1085.5486 (50) [M−H]⁻,<br>923.4966 (100) [M−Hex−H]⁻	—	—
**53**	911, 13.98 min	C47H76O17	0.9	1.94	100.00		911.5002 (100) [M−H]⁻	/70.9 eV/<br>911.50 (14) [M−H]⁻<br>749.45 (100) [M−Hex−H]⁻<br>603.39 (19) [M−Hex−dxHex−H]⁻<br>471.35 (22) [AGL−H]⁻ = [M−Hex−dxHex−Pen−H]⁻	ilexsaponin B2,<br>latifoloside A,<br>latifoloside B,<br>latifoloside D
**54**	911, 14.24 min	C47H76O17	1.2	2.04	46.60		911.4999 (100) [M−H]⁻	/70.9 eV/<br>749.45 (100) [M−Hex−H]⁻<br>471.35 (28) [AGL−H]⁻ = [M−Hex−dxHex−Pen−H]⁻	ilexsaponin B2,<br>latifoloside A,<br>latifoloside B,<br>latifoloside D
**55**	895, 14.40 min	C47H76O16	2.3	16.67	—	+	895.5040 (90) [M−H]⁻,<br>733.4520 (100) [M−Hex−H]⁻	/69.7. eV/<br>733.4552 (100) [M−Hex−H]⁻,<br>455.3517 (5) [AGL−H]⁻ = [M−Hex−dxHex−Pen−H]⁻	mateglycoside D,<br>matesaponin J3,<br>patriniaglycoside B-I,<br>patriniaglycoside B-II
**56**	895, 14.68 min	C47H76O16	3.3	7.16	5.15	+	895.5031 (90) [M−H]⁻,<br>733.4517 (100) [M−Hex−H]⁻	—	mateglycoside D,<br>matesaponin J3,<br>patriniaglycoside B-I,<br>patriniaglycoside B-II
**57**	953, 14.90 min	C49H78O18	3.9	31.11	—	+	953.5078 (82) [M−H]⁻,<br>791.4573 (100) [M−Hex−H]⁻	/74.3 eV/<br>749.45 (41) [M−Hex−Ac−H]⁻,<br>731.43 (76) [M−Hex−AA−H]⁻,<br>587.39 (100) [M−Hex−Hex−H]⁻,<br>455.35 (36) [AGL−H]⁻ = [M−Hex−Hex−Ac−Pen−H]⁻	acetylated 911, 13.98 min,<br>acetylated 911, 14.24 min
**58**	765, 15.24 min	C41H66O13	0.8	—	24.32		765.4424 (100) [M−H]⁻	—	—
**59**	765, 15.52 min	C41H66O13	1.6	—	4.52		765.4418 (100) [M−H]⁻	—	—
**60**	911, 15.77 min	C47H76O17	0.9	—	23.31		911.5002 (100) [M−H]⁻	—	—
**61**	911, 16.00 min	C47H76O17	1.9	—	27.35		911.4992 (100) [M−H]⁻	—	—
**62**	1219.58, 16.30 min	C58H92O27	−5.6	7.07	—		1219.5821 (100) [M−H]⁻	—	—
**63**	1219.58, 16.60 min	C58H92O27	5.7	6.21	—		1219.5837 (100) [M−H]⁻	—	—
**64**	895, 20.46 min	C47H76O16	0.9	6.17	39.89		895.5053 (100) [M−H]⁻	—	—
**65**	895, 20.59 min	C47H76O16	0.3	4.50	39.29		895.5058 (100) [M−H]⁻	—	—

No.	provisional name [pseudomolecular ion & retention time]	neutral formula	error [ppm]	RA [%] (Il.par.)	RA [%] (Il.aq.)	reported in [1]	MS mode fragments /30 eV/ (intensity%)	MS2 mode fragments /adjusted collision energy/	provisional identification based on *Ilex* saponins database and fragmentation pathway
**66**	749, 22.24 min	C41H66O12	1.0	9.83	—		749.4460 (100) [M−H]⁻	—	—

Abbreviations: AA—acetic acid loss, Ac—acetylation loss, AGL—aglycone, dxHex—deoxyhexose loss, Hex—hexose loss, HexA—hexuronic acid loss, Pen—pentose loss, RA—the relative area of peak, when the area of the largest one is calculated as 100%

Table S4 Fatty acid profile in leaves of *I. paraguariensis* and various varieties of *I. aquifolium* and *I. meserveae*.

Fatty acid[1]		RI Exp.[2]	RI Lit.[3]	*I. paraguariensis*	*I. aquifolium*			*I. meserveae*		
					Alaska	Ferox Argentea	Rubricaulis Aurea	Blue Angel	Blue Boy	Golden Girl
				Concentration (mg*g^{-1}) d.w						
Capric acid	C10:0	1057	1060	0.42	0.14	0.09	0.33	0.13	0.28	0.15
Lauric acid	C12:0	1201	1200	0.43	0.20	0.09	0.26	0.18	0.21	0.22
Myristic acid	C14:0	1402	1400	1.02	0.82	0.73	0.95	0.93	0.89	0.90
Myristoleic acid	C14:1ω5	1437	1436	0.38	0.23	0.13	0.35	0.21	0.31	0.26
Pentadecylic acid	C15:0	1499	1500	0.46	0.23	0.13	0.23	0.20	0.14	0.24
Palmitic acid	C16:0	1599	1600	18.08	12.05	15.03	12.66	14.03	13.04	12.51
Sapienic acid	C16:1ω10	1629	1628	0.73	0.41	0.30	0.21	0.22	0.15	0.18
Palmitoleic acid	C16:1ω7	1635	1632	2.36	0.99	3.19	1.16	2.14	0.92	1.01
Margaric acid	C17:0	1699	1700	0.59	0.32	0.14	0.32	0.22	0.28	0.39
Stearic acid	C18:0	1801	1800	6.39	2.37	4.17	2.50	4.12	2.56	2.60
Oleic acid	C18:1ω9	1817	1819	9.03	3.60	4.39	3.92	7.61	3.41	3.37
Vaccenic acid	C18:1ω7	1821	1824	1.96	0.55	1.82	0.64	1.28	0.50	0.59
Linoleic acid	C18:2ω6	1867	1874	4.23	8.40	5.84	8.72	5.63	8.21	10.08
α-Linolenic acid	C18:3ω3	1933	1928	5.92	21.31	9.72	19.28	9.09	19.61	22.16
Eicosatetraenoic acid	C20:4ω3	2101	2109	0.60	0.19	0.38	0.10	0.21	0.17	0.66
Arachidonic acid	C20:4ω3	2118	2115	2.71	0.71	9.11	0.54	1.84	0.40	0.58
Docosahexaenoic acid	C22:6ω3	2419	2416	1.67	0.53	1.73	0.66	0.93	0.45	0.66

[1] All compounds are expressed as GC-MS percentage of methyl esters; [2] experimental retention indices calculated against saturated fatty acids; [3] retention indices according to the Lipids Library 1.0;

1. Pachura, N.; Kupczyński, R.; Sycz, J.; Kuklińska, A.; Zwyrzykowska-Wodzińska, A.; Wińska, K.; Owczarek, A.; Kuropka, P.; Nowaczyk, R.; Bąbelewski, P., et al. Biological Potential and Chemical Profile of European Varieties of Ilex. *Foods* **2022**, *11*, 47.
